# Crystal Engineering as an Efficient Medicinal Chemistry Tool for Animal PK Bioavailability Enhancement in Early Pre-Clinical Research

**DOI:** 10.3390/ph19050803

**Published:** 2026-05-21

**Authors:** Axel Becker, Carolina von Essen, Lars Burgdorf, Marc Lecomte, Daniel Bischof

**Affiliations:** 1 Merck KGaA, Site Management—Lab Services, Frankfurter Strasse 250, 64293 Darmstadt, Germany; 2 Merck Healthcare KGaA, Global Research & Development, Frankfurter Strasse 250, 64293 Darmstadt, Germany; 3 Merck Life Science KGaA, Process Solutions, Frankfurter Strasse 250, 64293 Darmstadt, Germany

**Keywords:** co-crystal, crystal engineering, supersaturation, in vivo exposure, crystal structure, surface hydrophilicity

## Abstract

**Background:** A lean crystal engineering study was performed on the early pre-clinical POLθ inhibitor MSC178 to enable sufficient exposure for high-dose PK studies. **Methods:** COSMOquick-derived excess enthalpies in combination with a toxicological assessment of co-formers were used for the selection of four co-formers. Experimental crystallization trials were performed in a staged approach from a 15 mg scale, over a 50 mg upscale, to a final g-scale upscale of the most promising co-crystal form with 2,4-DHBA. **Results:** The 2,4-DHBA co-crystal form revealed more enhanced and sustained supersaturation plateaus in FaSSIF compared to the amorphous free base form, the 3,4-DHBA co-crystal form, and the 1,2-EDSA salt form. Moreover, the 2,4-DHBA co-crystal form was shown to be physically stable in the suspension vehicle for the PK study. The high physical stability toward physical-form conversion in the suspension vehicle as well as the more sustained supersaturation plateau in the non-sink dissolution profile could be attributed to the intrinsic features of the crystal structure as well as the assessed surface hydrophilicity of the co-crystal particles, both suggesting that rather hydrophobic surfaces are present that help preferentially attract stabilizing surfactants from the dissolution medium (taurocholate) and from the suspension vehicle (polysorbate, methocel), respectively. Successful upscale of the 2,4-DHBA co-crystal form was achieved in the small g-scale, revealing mainly isotropic crystal growth in primary particles as well as a pronounced tendency toward isotropically shaped dendrite-like secondary particles, both favored by a multi-dimensional hydrogen bonding network being present. Excellent agreement was shown for the extent of in vitro supersaturation behavior and in vivo exposure gain in the high-dose PK study for the 2,4-DHBA co-crystal form versus the amorphous free form. **Conclusions:** The co-crystal strategy can be successfully developed in early pre-clinical industrial research with lean methodologies to optimize sub-optimal phys.-chem. properties of a free base compound to achieve improved and less variable in vivo exposure between animals in high-dose PK studies.

## 1. Introduction

New chemical entity (NCE) drugs have represented the majority of approved drugs for the last 20 years and have also remained their backbone in recent years based on FDA approvals [1]. However, poor aqueous solubility is a recurring and even growing trend in current NCE discovery and development. Alongside these in vitro constraints, limitations in oral bioavailability for in vivo studies are typically observed [2,3]. These challenges are further intensified for a new class of beyond-rule-of-five drugs such as PROTACs [4]. Although i.v. administration of parenteral API solutions may be pursued to achieve the highest possible plasma levels, early in drug discovery and development, it is generally preferred to provide orally bioavailable delivery forms for animal PK studies as well, as i.v. administration has even higher demands on compound properties (such as solubility). Typical approaches to overcome solubility limitations of NCEs to achieve this are targeted in early formulation development, such as, among others, crystal engineering, self-emulsifying drug delivery systems (SEDDSs), and amorphous solid dispersions (ASDs) obtained by spray-dried dispersions (SDDs) or hot-melt extrusion (HME) processes [2,5]. However, these tools typically require systematic experimental screening and are typically done in the late research or early development stage, where larger compound quantities and more extended development timelines are ensured to allow for this.

In early MedChem research, in vivo profiling of candidates is typically done by vehicle screenings comprising solubility-enhancing excipients such as cyclodextrins, surfactants, or organic co-solvents. However, such rather generic technologies may not suffice for poorly soluble compounds to achieve solution vehicles at a desirable concentration, whereas suspension vehicles can lack sufficient exposure for poorly soluble compounds. In such cases, suspension vehicles based on optimized solid-state forms from crystal engineering approaches may be utilized as alternatives [6].

Here, we report a lean crystal engineering study to overcome such solubility constraints for the novel Polθ polymerase-domain (POLθ) inhibitor MSC178, providing an orally bioavailable tool compound for this class of compounds to study in vivo exposure. DNA polymerase θ is a central mediator of microhomology-mediated end-joining (MMEJ) and represents a promising synthetic lethal target in tumors with defects in homologous recombination and other DNA damage response (DDR) pathways. Defects in canonical DNA damage response pathways such as non-homologous end-joining (NHEJ), Fanconi anemia (FA), and homologous recombination (HR) can create a strong dependency on Polθ-mediated repair, thereby establishing a synthetic lethal relationship between Polθ and these DDR pathways. Polθ is upregulated in several human malignancies, including breast, ovarian, lung, liver and brain cancers, and elevated Polθ expression correlates with poor prognosis in, for example, lung cancer, soft tissue sarcoma, breast cancer and glioma [7,8,9,10].

**Figure 1:**
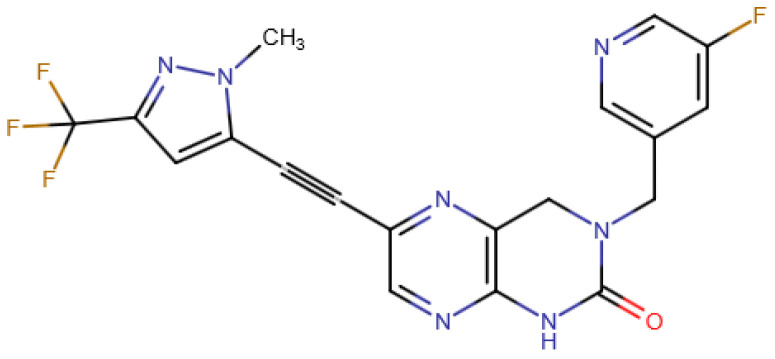
Chemical structure of MSC178.

The POLθ inhibitor MSC178 represents a very weak base, with a basic *pK_a_* of 2.6, which can most likely be attributed to the methyl-pyrazole nitrogen. This makes salt formation in principle feasible; however, strong acids are needed. The compound is rather lipophilic, with a log *P* of 3.0. All physicochemical properties are within the rule-of-five structural space [11]. The molecular structure of MSC178 is displayed in Figure 1. Its corresponding physicochemical properties are summarized in Table 1.

Vehicle screening was performed with the amorphous form of MSC178A (free form) in a range of generic solubilizing media, with the aim of obtaining a solution vehicle for p.o. administration in high-dose PK studies with the desired vehicle solution concentrations of 1 mg/mL and 5 mg/mL:

- Kolliphor HS 15 in water, 20%;
- PEG-400 in water, 20%;
- Glycofurol in water, 20%;
- Kleptose (HPB) in water, 20%, and Kleptose (HPB), 40% in water;
- Captisol in water, 20%;
- Tween-80, 2%, and Kolliphor EL, 12% in water;
- Kolliphor P 188 in water, 10%;
- NMP in PEG-300, 10%.

None of these solubilizing vehicles yielded a visually clear solution even at a lower targeted concentration of 1 mg/mL. All vehicles were subsequently diluted 1:1 with a blank vehicle to yield a solution concentration of 0.5 mg/mL; however, under these diluted conditions, no vehicle yielded a visually clear solution. Consequently, MSC178A (free form, amorphous) is insufficiently soluble (<0.5 mg/mL) for solution vehicles for the intended high-dose PK study in a broad range of solubilizing vehicles.

Different formulation options were considered as alternatives to solution vehicles for the planned high-dose PK study, under consideration of the required compound needs of the MSC178A free form for feasibility assessment, the time needed for lab-based feasibility options, and the anticipated probability of success in obtaining a formulation with improved exposure in high-dose animal PK studies. Among these options, nanosuspension of the free form was ruled out due to high compound need and low probability of success (compound anticipated to be solubility-limited as opposed to dissolution-rate limited according to DCS classification [12]), amorphous solid dispersion was discarded due to prolonged turn-around times, and advanced vehicle screening was not pursued due to an expected low probability of success to achieve a solution vehicle with the required concentration levels. A lean crystal engineering approach was selected as the most viable option to meet the low compound requirement for the feasibility study, quick turnaround time for the feasibility study, and a reasonable expected probability of success to improve exposure based on the literature data [6,13]. Identified alternative solid-state forms were intended for the assessment of suspension vehicle stability.

Whereas conceptional studies on co-crystal formation for improving solubility and in vivo exposure are widely published mainly on model compounds [6,14,15], to the best of our knowledge, this is one of the rare publications to show that a miniaturized and lean crystal engineering approach is a powerful technology to provide an efficient design of fit-for-purpose solid-state forms with improved in vivo exposure for in vivo profiling of drug candidates in early pre-clinical research from the pharmaceutical industry.

This article further outlines the specific benefits of co-crystal forms versus salt forms of the same API structure to achieve enhanced in vitro solubility and supersaturation, in combination with improved physicochemical properties such as physical stability (as also highlighted by Cavanagh et al. [16]).

The study also provides valuable insights into why some crystal-engineered forms are capable of achieving in vitro and in vivo supersaturation, whereas other forms from crystal engineering fail to do so.

Finally, this article illustrates how PAT-supported lab-scale upscaling can be efficiently utilized in early pre-clinical research for controlled manufacturing of g-scale quantities for comprehensive PK studies.

## 2. Results

All data and results reported here are based on POLθ inhibitor MSC178 as an exemplary drug candidate in the early research phase with a challenging physicochemical properties profile, as initially outlined.

### 2.1. Lean Crystal Engineering of MSC178A

#### 2.1.1. In Silico Assessment for Suitable Co-Formers for Salts and Co-Crystals

The selection of the most promising co-formers for co-crystal formation was based on excess enthalpy calculation in hetero-dimers (co-former versus MSC178) via COSMO-RS theory performed in COSMOquick software [17]. To accomplish a lean screening design, only highly ranked co-formers that were pre-aligned with the Merck Healthcare internal toxicology department were considered for experimental studies:

- Hydrochloric acid is ubiquitously present as an endogenous agent.
- 1,2-Ethanedisulfonic acid is part of the pre-aligned Merck Healthcare internal acid list for salt formation and can be found as a counter-ion in FDA Orange Book analysis [18].
- 2,4-Dihydroxybenzoic acid is a degradation product of cyanidin glycosides from tart cherries in cell cultures [19] and a metabolite found in human plasma after cranberry juice consumption [20].
- 3,4-Dihydroxybenzoic acid is a major metabolite of antioxidant polyphenols found in green tea [21].

The results of excess enthalpy calculations for selected co-formers are shown in Table 2. All four co-formers, regardless of salt formation or co-crystal formation scope, exhibit reasonably negative calculated excess enthalpies in dimeric interactions with MSC178. Hence, crystallization of a tailored mixed crystal form with these co-formers was deemed promising, and all four co-formers were selected for the first co-crystallization trials.

Looking at the rank order of excess enthalpies, the strongest dimeric interaction is suggested between MSC178A and 1,2-EDSA. As sulfonic acids bear highly polarized functional groups, this appears plausible, as the COSMO theory-calculated interaction strength is based on electrostatic surface charge profiles (*σ*-profiles) and the interaction of the most polarized surface charge spots [22]. For the same reason, a slightly stronger interaction between 2,4-DHBA and 3,4-DHBA is reasonable, as the hydroxy group in the alpha position to the carboxylic acid function in 2,4-DHBA allows an additional intramolecular hydrogen bond, resulting in an overall more polarized functional group (also seen at a more acidic *pK_a_* in 2,4-DHBA).

#### 2.1.2. Miniaturized Salt and Co-Crystal Formation Trials

To apply a cooling crystallization process for co-crystal formation, a condensed solubility assessment was made for MSC178A (free form, amorphous) and both dihydroxybenzoic acid co-formers intended for co-crystal formation. Solubility was assessed in three organic process solvents (acetonitrile, 1,4-dioxane, ethyl acetate) with medium polarity to allow sufficient solubilization of the lipophilic API compound as well as more hydrophilic co-formers (hence excluding alcohols as too-polar solvents and aromatic solvents as toonon-polar solvents). The results of the solubility assessment are shown in Table 3.

Based on solubility data, acetonitrile and ethyl acetate were selected as crystallization solvents, whereas 1,4-dioxane was deemed as a too-good solvent for MSC178A as well as for the co-formers. The significantly higher solubility of MSC178A in 1,4-dioxane compared with acetonitrile and ethyl acetate can be explained by the fact that dioxane has the lowest polarity among the three solvents. The fact that 1,4-dioxane also exhibits the best solubilization for both co-formers may be attributed to its hydrogen bonding potential with carboxyl acid groups [24]. The higher solubility of both dihydroxybenzoic acid co-formers in ethyl acetate compared with acetonitrile can be attributed to the greater number of hydrogen bonding options between the co-formers and ethyl acetate.

An initial cooling crystallization panel was run at the 15 mg scale (quantity of MSC178A), with the results summarized in Table 4. New crystalline forms were obtained from all initial co-crystallization experiments. Samples from acetonitrile exhibited more favorable properties for HCl, 2,4-DHBA, and 3,4-DHBA, as crystallizations from ethyl acetate for these co-crystals yielded increased incorporation of ethyl acetate in the crystal lattice or the formation of acetic acid in the case of HCl. Also, strongly over-stoichiometric ratios of co-formers were retrieved in ethyl acetate trials with 2,4-DHBA and 3,4-DHBA. Therefore, acetonitrile was selected as the preferred solvent for a small upscale to the 50 mg scale for these three co-crystals. Based on superior crystallinity in the obtained co-crystal form, co-crystallization with 1,2-EDSA was preferred from ethyl acetate, which was consequently selected as the solvent for a small upscale of the 1,2-EDSA co-crystal/salt.

To account for slight deviations in the retrieved stoichiometric ratios compared to the expected ratios for the 1,2-EDA co-crystal (0.7 eq. EDSA retrieved) and the 3,4-DHBA co-crystal (1.3 eq. DHBA retrieved), the 50 mg upscale trials were slightly amended regarding co-former quantities to target a mono-co-crystal form for 3,4-DHBA and both a hemi- and a mono-salt form for 1,2-EDSA (see Section 4 for details). The results of cooling crystallization upscales at the 50 mg scale (quantity of MSC178A) are summarized in Table 5. The 50 mg upscale experiments yielded highly crystalline co-crystal/salt forms with reasonable stoichiometric ratios (with max. deviation of 0.1-0-2 eq. of co-former equivalents) and no hints of excess free co-former crystallizing as a phase mixture. For 1,2-EDSA salts, both a mono-salt (form Ed-2) and a hemi-salt (form Ed-3) could be obtained by adjusting the quantities of sulfonic acid in the reaction. A HCl salt form, 1,2-EDSA mono-salt form, 2,4-DHBA co-crystal form and 3,4-DHBA co-crystal form were successfully reproduced at the 10 mg scale. All forms, apart from the HCl salt form Cl-1, exhibited very low residual solvent levels. The HCl salt form Cl-1 exhibited a slightly increased residual acetonitrile level; however, it was still it was still below the stoichiometric levels expected for a solvate form. This is probably due to some strong solvent entrapment between particles. All 50 mg samples were selected for further profiling.

### 2.2. Solid-State Characterization Data of MSC178 Forms

For selected solid-state forms [1,2-EDSA hemi-salt form Ed-3, 2,4-DHBA co-crystal form 24-1, 3,4-DHBA co-crystal form 34-1], solid-state IR-spectra were recorded in comparison with the free base amorphous form; however, no clear conclusions regarding salt form (protonation of API scaffold) versus co-crystal (no protonation) can be deduced from the spectra. Based on the *pK_a_* values of API and involved acids, salt formation can be assumed as a solid-state form in the case of HCl and 1,2-EDSA, and co-crystal formation can be assumed as a solid-state form in the case of 2,4-DHBA and 3,4-DHBA, in line with FDA guidance for differentiation of salt versus co-crystal forms [25]. In the case of the 2,4-DHBA form 24-1 and the 3,4-DHBA form 34-1, this is also confirmed by Single-Crystal X-Ray Diffractometry and electron diffraction data, as shown below.

**Figure 2:**
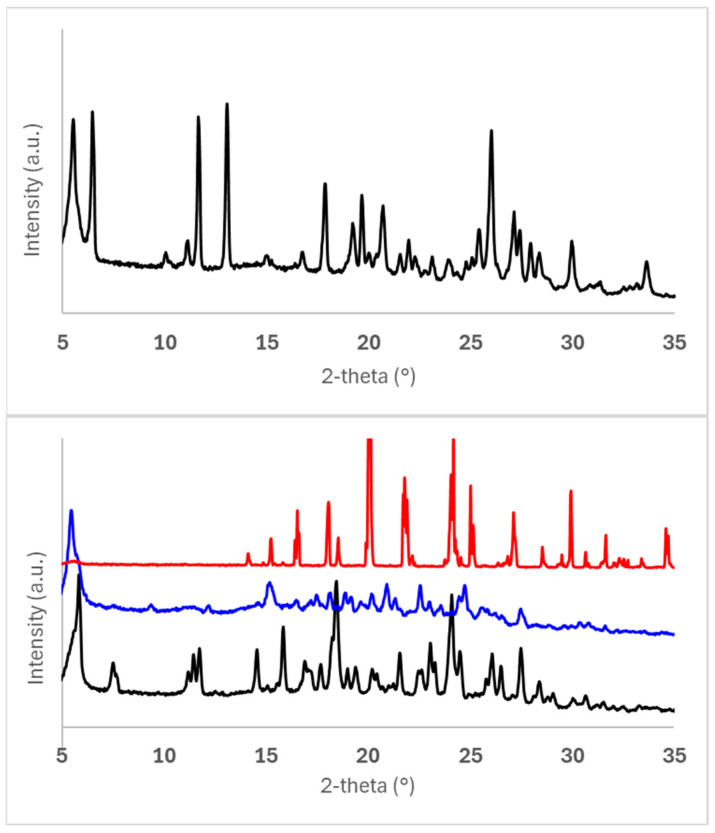

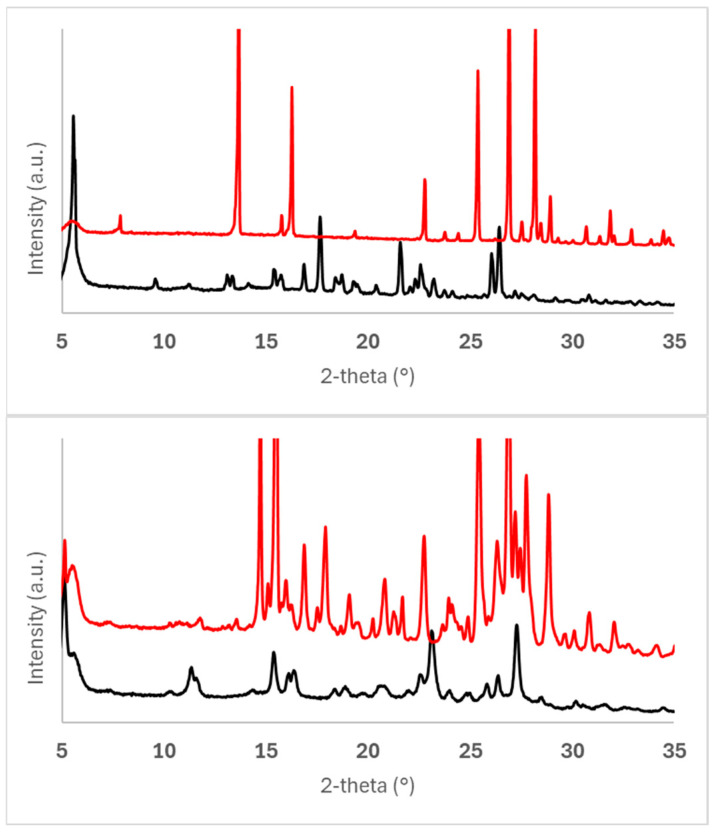
PXRD patterns of co-crystal/salt forms of MSC178. (**Top**): HCl salt form Cl-1. 1,2-EDSA salt forms Ed-2 (black) and Ed-3 (blue) versus 1,2-EDSA co-former (red). (**Middle**): 2,4-DHBA co-crystal form 24-1 (black) versus 2,4-DHBA co-former (red). (**Bottom**): 3,4-DHBA co-crystal form 34-1 (black) versus 3,4-DHBA co-former (red).

PXRD patterns of the 50 mg scale samples, as outlined in Table 5, are shown in Figure 2. All co-crystal/salt forms can unequivocally be differentiated and identified by their characteristic PXRD profiles. No hints of the presence of excess free acid co-formers are observed in the case of forms Ed-2, Ed-3, 24-1, and 34-1. The absence of pronounced underlying broad background halos confirms that no significant amorphous fractions are present in any form. Signal intensities and the signal/noise ratio in 1,2-EDSA hemi-salt form Ed-3 are lower than in the other forms due to the smaller sample quantity used in the X-ray preparation. Although all forms can be deemed highly crystalline based on the multitude of sharp signals observed, slightly broader peak widths in the PXRD data suggest that 1,2-EDSA hemi-salt form Ed-3 and 3,4-DHBA co-crystal form 34-1 may exhibit a slightly reduced degree of crystallinity.

**Figure 3:**
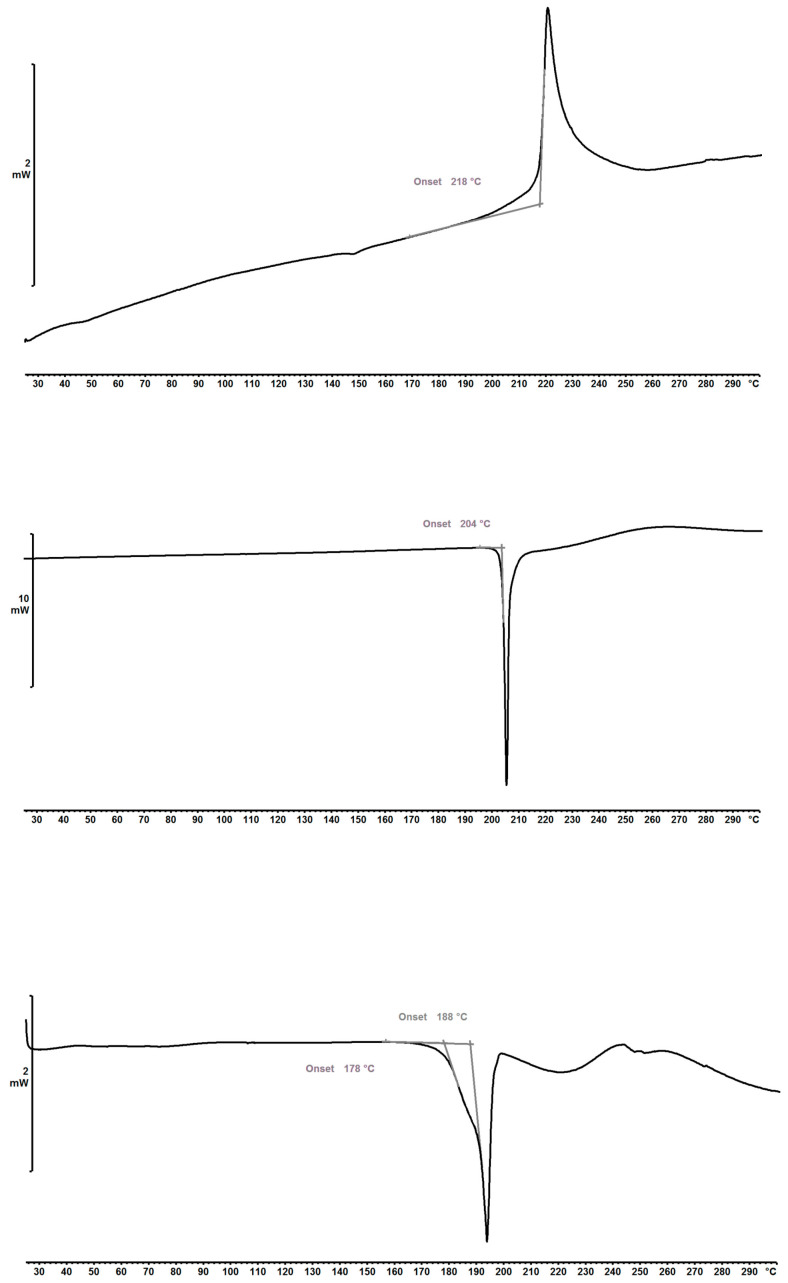
DSC heating traces (exo upwards) of co-crystal/salt forms of MSC178. (**Top**): 1,2-EDSA hemi-salt form Ed-3. (**Middle**): 2,4-DHBA co-crystal form 24-1. (**Bottom**): 3,4-DHBA co-crystal form 34-1.

DSC heating scans of the 1,2-EDSA hemi-salt form Ed-3, 2,4-DHBA co-crystal form 24-1, and 3,4-DHBA co-crystal form 34-1 are shown in Figure 3. The 2,4-DHBA co-crystal form 24-1 exhibits a melting point onset of approx. 203 °C, whereas the 3,4-DHBA co-crystal form 34-1 exhibits an overlapped melting peak with an initial onset at approx. 178 °C and an extrapolated second onset of an overlapped main peak at approx. 188 °C. The broader and overlapping melting peak in the 3,4-DHBA co-crystal form can be linked to the slightly decreased degree of crystallinity as deduced from PXRD peak widths. Overall, the significantly higher melting point of the 2,4-DHBA co-crystal may indicate a stronger crystal lattice interaction compared to the 3,4-DHBA co-crystal. In contrast to both co-crystal forms, the 1,2-EDSA hemi-salt form Ed-3 exhibits no clear melting peak, but rather an exothermic peak at approx. 218 °C. This can be attributed to thermal decomposition upon heating; hence, it can be concluded that the hemi-edisylate salt form exhibits a virtual melting peak higher than 218 °C. The highest thermal stability in this solid-state form, despite hints of slightly reduced crystallinity as suggested by the PXRD line widths, can be explained by significantly stronger lattice interactions due to ionic interactions in the salt form compared with hydrogen bonding interactions in the co-crystal forms.

**Figure 4:**
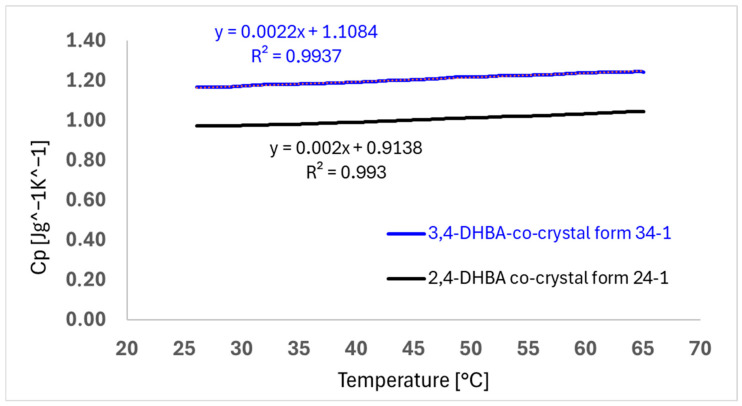
Heat capacity (Cp) versus temperature profiles of 2,4-DHBA co-crystal form 24-1 (black) and 3,4-DHBA co-crystal form 34-1 (blue) of MSC178 (mean of 2 samples of each co-crystal form, each measured with *n* = 2; overall mean standard deviation per form: 0.11-0.14 J/g×K).

For further in-depth characterization of both obtained co-crystal hits, the 2,4-DHBA co-crystal form 24-1 and the 3,4-DHBA co-crystal form 34-1 were profiled by temperature-modulated DSC (mDSC) to determine heat capacities *Cp* as a measure for solid-state mobility of molecules in the crystal lattice. The resulting heat capacity profiles as a function of temperature are shown in Figure 4. It can be seen that the 2,4-DHBA co-crystal form 24-1 exhibits lower heat capacities in the investigated temperature range of 25–65 °C (0.97–1.07 J/g×K) compared to the 3,4-DHBA co-crystal form 34-1 (1.17–1.24 J/g×K); however, these differences are at best borderline significant based on the observed scattering of data derived from standard deviations (0.11–0.14 J/g×K) within multiple determinations per form. Nevertheless, as a trend, form 24-1 appears to exhibit a lower heat capacity and consequently also a lower solid-state-related molecular mobility. This suggests a stronger overall coordination of molecules in the crystal lattice of the 2,4-DHBA co-crystal form 24-1 compared to that in the 3,4-DHBA co-crystal form 34-1. Moreover, form 34-1 also appears to exhibit a very slightly steeper slope in heat capacity changes upon heating, suggesting a slightly stronger solid-state entropy contribution [26]. The heat capacity data fit well with the observed higher melting point of form 24-1.

**Figure 5:**
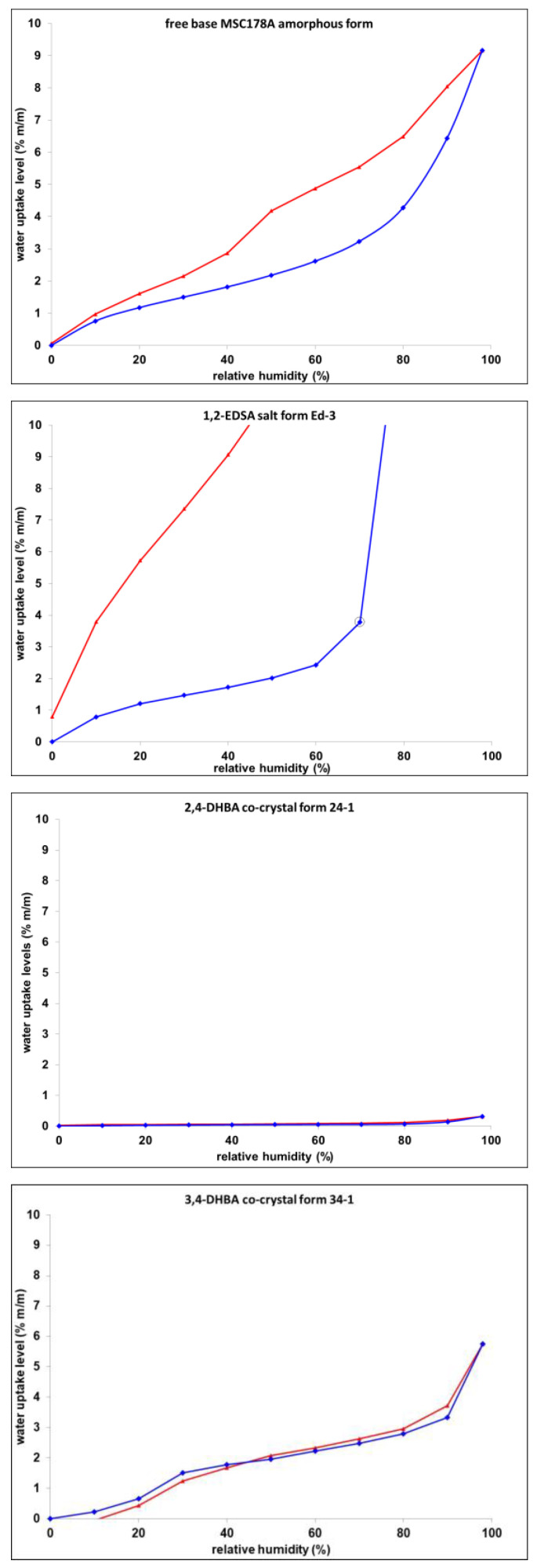
Water vapor sorption isotherms (25 °C) of co-crystal/salt forms of MSC178 (blue diamonds: adsorption segment 0–98% rh; red triangles: desorption segment 98–0% rh). (**Top**): Free base MSC178A amorphous form (for comparison). (**2nd from top**): 1,2-EDSA salt form Ed-3 ^1^. (**2nd from bottom**) 2,4-DHBA co-crystal form 24-1. (**Bottom**) 3,4-DHBA co-crystal form 34-1; ^1^ y-scale limited to 0–10% (m/m) for comparison purposes.

Water vapor sorption isotherms were acquired at 25 °C for the 1,2-EDSA hemi-salt form Ed-3, 2,4-DHBA co-crystal form 24-1, and 3,4-DHBA co-crystal form 34-1 to assess their hygroscopicity behavior compared to the free base amorphous form. Respective data are shown in Figure 5. The amorphous form of the free base exhibits a pronounced water uptake throughout the entire rh range and a strong and broad hysteresis upon desorption. This can be attributed to strong contributions of bulk absorption effects into the amorphous matrix. The 2,4-DHBA co-crystal form 24-1 exhibits a very flat and fully reversible sorption profile, with a maximum water uptake below 0.5% (m/m), characteristic of solely surface-attached water uptake due to physisorption processes. In contrast to this, the 3,4-DHBA co-crystal form 34-1 exhibits a more pronounced water uptake profile; however, it is still reversible in nature. The pronounced but reversible uptake levels in combination with the characteristic strong water uptake step at low rh levels may be indicative of channel-hydrate sorption behavior. 1,2-EDSA hemi-salt Ed-3 exhibits by far the strongest water uptake at elevated rh levels > 70% rh. The sorption steps do not reach an equilibrium stage, indicative of deliquescence processes taking place. Although no indication for a hydrate formation step is seen in the lower rh range of 0–60% rh, the hemi-salt form exhibits pronouncedly higher uptake sorption levels in this humidity range compared to the 2,4-DHBA co-crystal form 24-1. This may be explained by a higher attraction of water vapor to particle and crystal surfaces due to ionic interactions in the salt crystal lattice, which can trigger water attraction in coordination with ionic centers (e.g., as ion-associated hydrates) [27].

To derive a more quantitative description of hydrophilic versus hydrophobic surface domains present on the crystal faces of the 2,4-DHBA-co-crystal form 24-1, 3,4-DHBA co-crystal form 34-1, and 1,2-EDSA salt form Ed-3, water vapor sorption profiles and n-octane organic vapor sorption data were acquired at higher partial pressure resolution in the partial pressure range of 5–50%. As the BET sorption model is not valid for water vapor sorption data (due to gas–gas interactions and cluster adsorption), a water monolayer equivalent coverage was determined based on the sorption equivalent of the strongest interaction according to the Excess Surface Work (ESW) sorption model [28] and converted into a water-specific surface area equivalent as a quantitative measure for surface hydrophilic domains. For assessment of the hydrophobic surface area in the same samples, the BET sorption model was applied on n-octane gravimetric vapor sorption [29]. Water vapor sorption data based on the ESW model and n-octane sorption data based on the BET model for the 1,2-EDSA salt, 2,4-DHBA co-crystal, and 3,4-DHBA co-crystal in comparison with the free base amorphous form are available in the Supplementary Materials in Figure S1 (water vapor sorption plots, ESW model) and Figure S2 (n-octane sorption plots, BET model), respectively. The resulting surface characterization data are summarized in Table 6.

The sorption data reveal that the 2,4-DHBA co-crystal exhibits a pronouncedly lower hydrophilic surface domain compared to the 1,2-EDSA salt, both in total water-specific surface area equivalent and in the relative surface hydrophilic ratio. The 3,4-DHBA co-crystal form 34-1 exhibits the pronouncedly highest total water-specific surface area equivalent compared to the other forms; however, when looking at the surface hydrophilic ratio, the form 34-1 lies somewhat in between the apparently more hydrophobic 2,4-DHBA co-crystal form 24-1 and the apparently more hydrophilic 1,2-EDSA salt form Ed-3. The highest total water-specific surface area equivalent may be explained by the assumed channel structure in this form, as indicated by the full water vapor adsorption/desorption isotherms and the crystal structure. The surface hydrophilic ratios suggest that both co-crystal forms have overall less hydrophilic surface chemistry in their crystal planes compared to the salt form; however, pronounced differences in surface hydrophilicity can still be observed among different co-crystal structures. It is interesting to note that the free base amorphous form, which was investigated for comparison, exhibits an apparently higher surface hydrophilic ratio than the 2,4-DHBA co-crystal form 24-1 despite not bearing any polar co-former/counter-ion in the solid-state structure. Here, potential contributions from bulk absorption effects (as opposed to mere surface adsorption effects) may play a role as well. As the 1,2-EDSA salt and the 3,4-DHBA co-crystal reveal similar high or higher surface hydrophilic ratios compared to the amorphous free base (as one would expect for such mixed-crystal systems with polar co-formers), this shows the extraordinarily low surface hydrophilicity in the 2,4-DHBA co-crystal structure.

The observed variability in the derived surface area data is low to moderate for water vapor sorption-derived surface area data (deviations for single versus mean values in the range 0.3–10%) and partly enhanced for n-octane-derived surface area data (deviations for single versus mean values in the range 0.2–24%). The resulting surface hydrophilic ratio levels exhibit single versus mean deviations in the range of 2.9–33%. Despite the partly enhanced scattering within the duplicate determinations, the observed differences in surface properties can be considered significantly different, especially when differentiating the most hydrophobic form 24-1 versus the medium hydrophobic forms 34-1 and the amorphous free base versus the most hydrophilic form Ed-3. Therefore, the sorption data strongly suggest differences in surface chemistry between salt and co-crystal forms in a more quantitative and illustrative manner.

**Figure 6:**
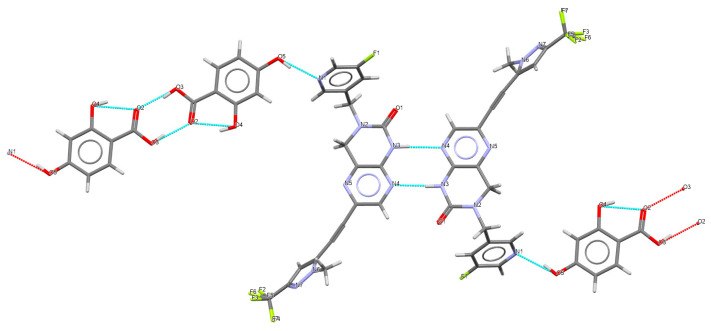
Hydrogen bond network from crystal structure solution of 2,4-DHBA co-crystal form 24-1.

**Figure 7:**
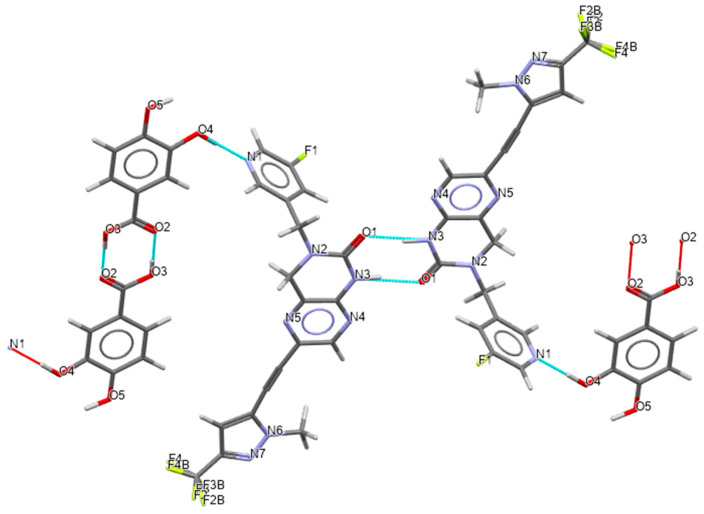
Hydrogen bond network from crystal structure solution of 3,4-DHBA co-crystal form 34-1.

**Figure 8:**
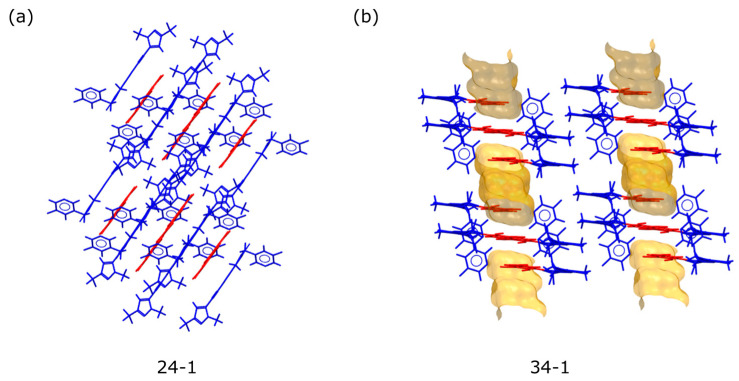
Packing arrangements and voids (yellow) in the crystal structures of the (**a**) 2,4-DHBA and (**b**) 3,4-DHBA co-crystals. API molecules are represented in blue, while co-former molecules are depicted in red.

Crystal structures could successfully be solved for the 2,4-DHBA co-crystal form 24-1 and the 3,4-DHBA co-crystal form 34-1. Residual electron density clearly revealed the correct hydrogen positions, confirming the presence of a co-crystal form. The hydrogen bonding network of the 2,4-DHBA co-crystal form 24-1 is shown in Figure 6. The parent molecule forms homo-synthons through interactions between two 3,4-dihydro-1H-pteridin-2-one moieties (N3-H…N4). Additionally, two 2,4-dihydroxybenzoic acid (2,4-DHBA) molecules interact via carboxylic acid–carboxylic acid interactions. These homo-synthons are interconnected by hydrogen bonds between the para-hydroxyl group of 2,4-DHBA and the nitrogen N1 of pyridine, resulting in one-dimensional chains. The ortho-hydroxyl group on 2,4-DHBA forms an intramolecular hydrogen bond (OH hydroxyl…OC carboxyl), which aligns with one of Etter’s principles: “six-membered-ring intramolecular hydrogen bonds preferentially form over intermolecular hydrogen bonds.” [30]. Furthermore, the crystal structure is stabilized by aromatic interactions between pyrazine and benzene, as well as between adjacent pyrazole moieties. The hydrogen bonding network of the 3,4-DHBA co-crystal form 34-1 is shown in Figure 7. The structure contains channels surrounded by the hydroxyl groups of 3,4-DHBA along the crystallographic *a*-axis. This confirms the observations made in water vapor sorption profiles. The hydrogen bonding motifs are remarkably similar compared to form 24-1. Homo-synthons between the two parent molecules as well as the co-former are observed, with the parent being connected through amide–amide (N3-H…O1) interactions. The co-former is connected by hydrogen bonds between the meta-hydroxyl group and the pyridine nitrogen. The para-hydroxyl group of the acid does not show any hydrogen bonding but is very close to the void channels, presumably interacting with water molecules. The packing arrangements of the crystal structures are shown in Figure 8. It can be seen that in form 24-1, the co-former homo-synthons are enclosed by API molecules, while in form 34-1, adjacent chains of API and co-crystal molecules with voids running in parallel are formed.

**Figure 9:**
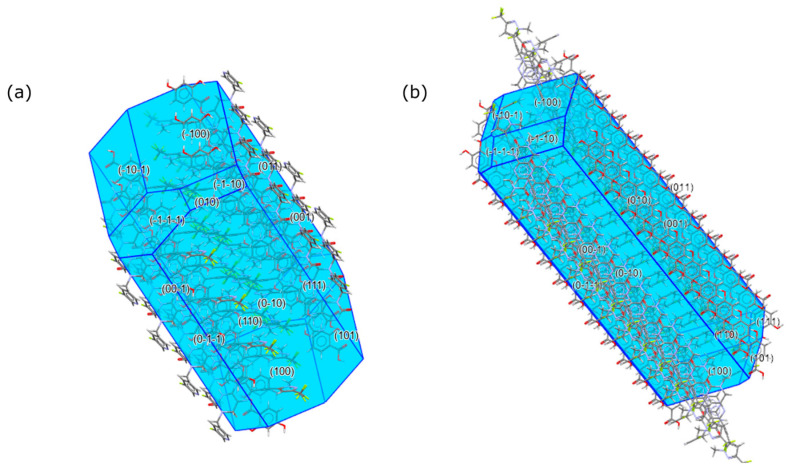
BFDH-predicted morphology of the (**a**) 2,4-DHBA and (**b**) 3,4-DHBA co-crystals, including molecular packing. Facets are presented by their Miller indices in round brackets.

Crystal morphologies of the 2,4-DHBA co-crystal and the 3,4-DHBA co-crystal were computed using the Bravais–Friedel–Donnay–Harker (BFDH) method [31] included in the latest release of the visualization software package [32]. It calculates approximate crystal morphology based on crystallographic geometrical considerations, including the unit cell shape and symmetry operator information. The BFDH assessment is illustrated in Figure 9 and indicates that hydrophobic pyrazole and fluoropyridines are seen in the main faces of the crystal of form 24-1, whereas the dominant faces of form 34-1 exhibit polar hydroxyl groups.

### 2.3. In Vitro Biopharmaceutical Assessment of Crystal Forms

#### 2.3.1. Non-Sink Miniaturized Dissolution Assessment

All successfully obtained co-crystal and salt forms from the 50 mg upscale experiment were profiled for potential improvement in dissolution behavior compared to the amorphous free form under non-sink conditions in biorelevant intestinal medium (FaSSIF) to mimic in vivo conditions [33].

**Figure 10:**
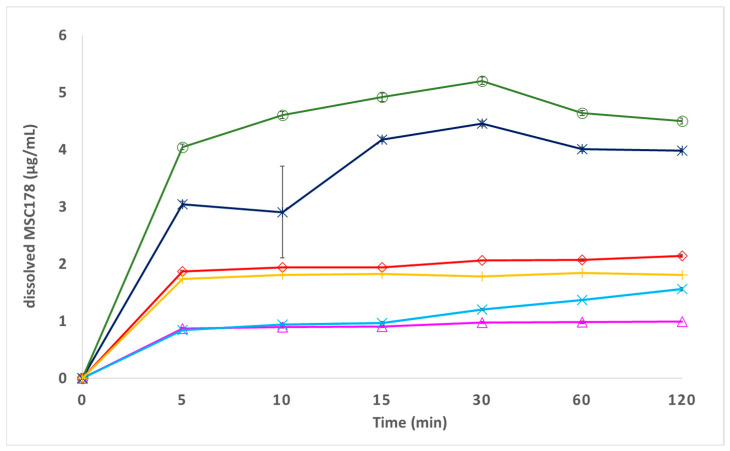
Non-sink dissolution profiles of solid-state forms of MSC178 in FaSSIF (pH 6.5), 37 °C (mean from *n* = 2 ± deviations single versus mean values from duplicate determination). Green circles: 2,4-DHBA co-crystal form 24-1. Dark blue stars: 1,2-EDSA hemi-salt form Ed-3. Red diamonds: free base MSC178A amorphous form. Orange dashes: 3,4-DHBA co-crystal form 34-1. Light blue crosses: 1,2-EDSA mono-salt form Ed-2. Magenta triangles: HCl salt Cl-1.

The resulting non-sink dissolution profiles are displayed in Figure 10.

Apart from a single data point at 10 min for form Ed-3, which exhibits enhanced variability within the duplicate determination, all determined dissolution levels exhibit very low scattering within the duplicate determination (deviations for single versus mean values below 0.1 µg/mL for all other data points). This shows that despite the low levels of dissolved MSC178 and despite the low number of replicates, the differences in non-sink dissolution profiles between different solid-state forms can be considered significant.

It can be seen that the 2,4-DHBA co-crystal form 24-1, as well as the 1,2-EDSA hemi-salt form Ed-3, exhibits enhanced levels of dissolved API upon non-sink dissolution compared to the amorphous free form (*Cmax* [30 min] level increased by a factor of 2 [hemi-salt form Ed-3] and a factor of 2.5 [co-crystal form 24-1], respectively). Moreover, this supersaturation effect appears to be stable up to 30 min at least, and plateau levels are established between 60 min and 120 min (a very slight decrease in dissolution levels from 30 min to 60 min is observed for both crystalline forms, which may be indicative of a starting precipitation of a less soluble parent form). The finding that these two crystalline forms exhibit higher disolution plateau levels compared to the amorphous free base suggests that the solubility constraints of the free form is not driven by lattice energy effects (no “brick-stone” compound), but rather by limitations in solubilization due to an insufficient hydration energy (“grease-ball” compound), also supported by a high log *P* value as a strongly contributing factor for poor solubility [34]. It must be noted that potentially confounding parameters that contribute to differences in non-sink dissolution behavior may include differences in particle size, surface area, wettability, or aggregation state (see discussion Section 3). The enhanced dissolution behavior of the edisylate salt form is likely driven by higher solubility of the protonated compound in the diffusion boundary layer [35]. However, the corresponding mono-edisylate salt form Ed-2 does not show any dissolution improvement, which may be impaired by the apparently higher crystallinity in the mono-edisylate salt compared to the hemi-edisylate salt form. It is also interesting to note that no enhanced dissolution levels are observed for the alternative co-crystal form 34-1 with 3,4-DHBA as co-former, despite the apparent hint of a slightly reduced crystallinity in this form compared to the 2,4-DHBA co-crystal form 24-1. The HCl salt form Cl-1 exhibits the lowest dissolution level among all forms, which can be explained by a solubility decrease due to the common-ion effect of chloride ions from FaSSIF [35]. Although the HCl salt form appears to be the least soluble and hence the most stable form in such a medium with excess levels of dissolved chloride ions, none of the other forms shows a pronounced precipitation effect down to the lower dissolution levels of the HCl salt form, even up to 120 min in the dissolution medium. This may be inhibited by potential energy barriers to nucleation of the HCl salt form.

The observed differences in the in vitro dissolution data of the solid-state forms, specifically the higher levels of dissolved MSC178 in the crystalline forms 24-1 and Ed-3 versus the amorphous free base form and the crystalline form 34-1, are based on a non-sink dissolution set-up, where excess solid-state material is present at any time of the dissolution study. Although some differences between the solid-state forms are seen already in the build-up rates of dissolved levels in early timepoints of 5–15 min (which may, in principle, also be driven by potential differences in particle size), the data in Figure 10 clearly show that towards more extended timepoints of 60–120 min, plateau levels are observed for all forms. Moreover, the differences in the final plateau levels of the non-sink dissolution profiles between different solid-state forms are in fact more pronounced than the differences in early timepoint dissolution build-up. This suggests that the main effect for different solid-state forms is due to a solubility gain rather than kinetic dissolution effects, which is in line with the initial assessment of MSC178 as a DCS solubility-limited compound.

#### 2.3.2. Vehicle Stability Assessment of Lead Forms

**Figure 11:**
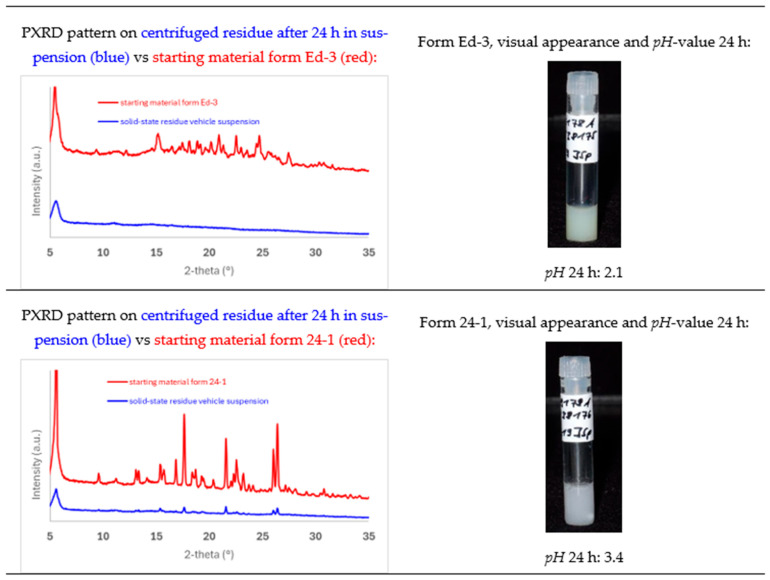
Stability assessment for co-crystal/salt forms in methocel/tween suspension vehicle (0.5% methocel, 0.25% tween-20 in water; target conc. 5 mg/mL free drug; unbuffered vehicle). (**Top**): 1,2-EDSA salt form Ed-3 ((**left**): PXRD residue; (**right**): visual appearance and *pH*). (**Bottom**): 2,4-DHBA co-crystal form 24-1 ((**left**): PXRD residue; (**right**): visual appearance and *pH*).

Based on the observed supersaturation levels in the non-sink dissolution assay, the 1,2-EDSA hemi-salt form Ed-3 and the 2,4-DHBA co-crystal form 24-1 were selected for in vivo high-dose PK studies for assessment of in vivo exposure from p.o. administration. A methocel/tween suspension (0.5% methocel, 0.25% tween-20 in water) was selected as the formulation vehicle. For this purpose, a physical stability assessment of both forms in the suspension vehicle was conducted with respect to sedimentation effects, as well as with respect to physical form conversion. The results of the suspension vehicle stability assessment are shown in Figure 11.

While both crystal-engineered forms yield a virtually stable suspension regarding sedimentation effects up to 24 h, only the 2,4-DHBA co-crystal form 24-1 is preserved as a crystalline solid-state form in the suspension, whereas the crystalline 1,2-EDSA hemi-salt form Ed-3 is shown to undergo phase conversion/salt disproportionation to an amorphous form (most likely the amorphous free form). The higher reactivity of the salt form towards disproportionation may be explained by two contributing factors when assuming that, to a very slight extent, dissolution of the solid-state form in the suspension vehicle will occur. Firstly, although the protonation effect can be expected to be preserved into the diffusion boundary layer of the immersed solid of the salt, the attained *pH* value in the diffusion layer likely does not reach a strongly acidic *pH* of 1.8 or lower (supported by the measured *pH* value of the final suspension of 2.1), which would be required to stay below the anticipated *pH-max* of the salt (i.e., the *pH* value below which the edisylate salt is thermodynamically stable, as typically, the *pH*-max of a salt is approx. 1–2 *pH* units below *pK_a_* [36]). And secondly, a higher local supersaturation in the diffusion boundary layer is likely attained for the salt form due to a stronger *pH* shift in the diffusion layer compared to the 2,4-DHBA co-crystal form, which then may trigger the precipitation of a less soluble free amorphous form (as the more stable entity above the assumed *pH-max* of the salt in the vehicle). Via such a mechanism, it is suggested that a subsequent conversion of the entire solid phase can occur over repeated equilibrium processes.

### 2.4. Process Upscale of the 2,4-DHBA Co-Crystal

A staged upscale approach was pursued for the 2,4-DHBA-co-crystal form 24-1 as the most promising solid-state form from the crystal engineering study (sustained supersaturation effect versus the amorphous free base; physically stable form in the suspension vehicle for the PK study):

- A small upscale at the 80 mg scale was performed to provide sufficient material for an initial high-dose PK study in mice.
- Based on the positive outcome of in vivo exposure assessment of the 2,4-DHBA co-crystal form 24-1 from the high-dose PK study in mice (see Section 2.5), a further small g-scale upscale was performed to provide sufficient material for comprehensive in vivo exposure behavior (e.g., dose escalation studies). To gain further understanding of the co-crystal formation step, the g-scale experiment was also monitored closely by Process-Analytical-Technology (PAT) tools in the g-scale reactor (in situ video-microscopy [ParticleViewTM]; in situ chord-length-distribution [ParticleTrackTM]).

The results of the upscale experiments for material provision of the PK studies are summarized in Table 7. PAT data from the g-scale upscale are available in Supplementary Materials Figure S3. The resulting chord length distribution profiles at the end of the 5 °C holding time of the g-scale upscale experiment are available in Supplementary Materials Figure S4.

Although the 80 mg scale process (multiple cooling/heating cycles 50–55 °C at 0.075 °C/min) and the 1.7 g scale process (single cooling ramp 60–65 °C at 0.1 °C/min) utilized different process settings, both upscale experiments yield a highly crystalline co-crystal form 24-1, with no hints of free excess 2,4-DHBA co-former being present (despite the stoichiometric ratio of co-former:API in the 80 mg sample being very slightly over-stochiometric). This indicates a good robustness of the co-crystal formation step, even without the use of seed crystals. The residual solvent levels of the crystallization solvent acetonitrile are well below the ICH limit, regardless of an additional washing step being utilized (1.7 g scale) or not (80 mg scale). PAT data from the 1.7 g scale experiment indicate that a clear solution is obtained at 60 °C upon heating, and spontaneous nucleation upon cooling is observed at approx. 57 °C. Thus, the metastable zone width is presumably rather narrow, posing a challenge for a controlled seeding strategy. Initially formed primary particles appear to exhibit a slightly elongated shape; however, upon further cooling, almost instantly, the formation of intergrown dendrite-like secondary particles is seen. Particle sizes of the 2,4-DHBA co-crystal final product are in the range of 10–100 µm based on square-weighted CLD data, with strongly isotropically shaped particles due to the intergrown secondary particles. As a result, the material exhibits an excellent filtration behavior.

### 2.5. In Vivo Oral PK Study of the 2,4-DHBA Co-Crystal in Mice

**Figure 12:**
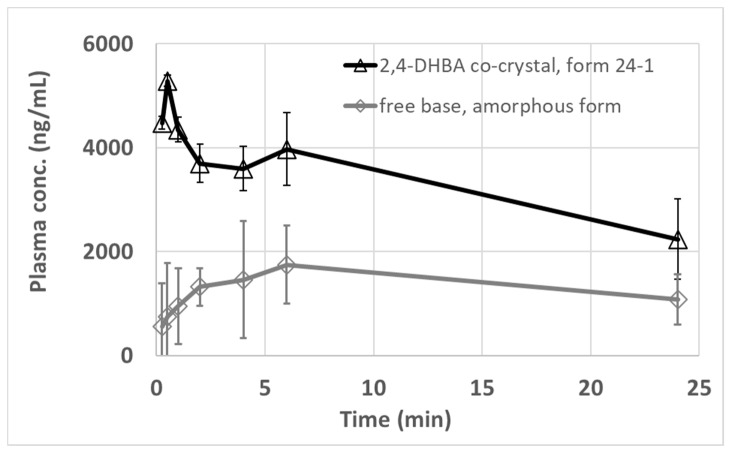
Plasma concentration–time profiles from the oral high-dose PK study in mice. (The suspension vehicle used was methocel/tween [0.5% methocel, 0.25% tween-20 in water], 5 mg/mL, 50 mg/kg free base; data points represent mean data ± SD from *n* = 3 animals for each form. Experimental details for the in vivo PK study and bioanalytical method used for MSC178 quantification can be found in the Materials and Methods Section.)

The results of the oral high-dose PK study in mice for the 2,4-DHBA co-crystal form 24-1 in comparison with the free base amorphous form are summarized in Table 8. The corresponding plasma concentration profiles over time are displayed in Figure 12.

The oral high-dose (50 mg/kg free base) PK data in mice displayed in Figure 12 and in Table 8 clearly show that the total exposure [*AUC*_0__–24 h_] observed after dosing as a suspension in methocel/tween [0.5% methocel, 0.25% tween-20 in water] of the 2,4-DHBA co-crystal form 24-1 is clearly higher (2.36-fold) than the amorphous free form of MSC178. The absorption kinetics and extent are also clearly different, with a rapid (0.5 h versus 6 h) *tmax* and higher *Cmax* (3.0-fold difference) observed for the 2,4-DHBA co-crystal form 24-1 as compared to the amorphous free form of MSC178. The data displayed in Figure 12 also show that the inter-animal variability in mice is smaller (error bars, *SD n* = 3) after oral dosing with the 2,4-DHBA co-crystal form 24-1 of MSC178 as compared to its amorphous free form.

## 3. Discussion

This study shows the interesting phenomenon that crystalline forms (either salt or co-crystal) dissolve faster and supersaturate more than the amorphous form of free bases. Such differences may, in principle, also be impacted by underlying differences in aspects such as particle size distribution, surface area, wettability, or aggregation state, as potentially confounding parameters. Although no direct assessment of particle size distribution data (e.g., via the laser diffraction method) was possible due to limitations in available sample quantities, the observed surface characterization data, as shown in the experimental section, reveal clearly higher surface area data for the amorphous form compared to the crystalline forms 24-1 and Ed-3 (both showing enhanced dissolution and supersaturation behavior), suggesting that higher non-sink dissolution levels of crystalline forms are not mainly driven by higher surface area and corresponding smaller particle sizes. Also, a more extended aggregation state in the amorphous form sample is not supported by the surface area data. Moreover, the co-crystal form 24-1 was shown to exhibit more hydrophobic surface domains compared to the amorphous form, suggesting no improved wettability for the co-crystal form 24-1 (whereas for the salt form Ed-3, the higher surface hydrophilicity compared to the amorphous form suggests that surface wettability may be a confounding factor). Although the surface area data suggest that the differences therein are not the key driver explaining non-sink dissolution behavior and plateau levels, controlled variations of such confounding parameters for each solid-state form in a more systematic manner would be desirable to fully understand these underlying confounding effects. However, a defined adjustment of particle size in solid-state forms would require extensive particle engineering activities as part of crystallization process development for each solid-state form, which was not in the scope of this study due to limitations in material quantities. Also, in contrast to particle size and surface area, parameters such as surface hydrophilicity and corresponding wettability are likely intrinsic features of the respective crystal structures and may not be subject to adequate control via the crystallization process.

In contrast to the common perception and the literature data showing higher solubility and dissolution effects of salts versus co-crystals [37,38], this study shows a higher and more sustained supersaturation effect for a co-crystal form compared to a salt form. A salt form can overcome solubility constraints by the protonation effect in the diffusion layer; however, for such weakly basic compounds, this is typically not a sustained effect and has a high risk of deprotonation in less acidic milieu [36,39], which could also be seen in the instability of the 1,2-EDSA salt in the formulation vehicle. Co-crystals generally do not offer the potential of an improved hydration layer interaction via protonated charged drugs in the diffusion layer. This could be one reason why the 3,4-DHBA co-crystal does not provide any dissolution plateau and supersaturation benefit versus the amorphous free form (or rather, a potentially improved solubilization benefit in the aqueous hydration layer may be so small that it just compensates for the additional energy barrier of lattice energy in the 3,4-DHBA co-crystal versus the amorphous free form). In contrast, the 2,4-DHBA co-crystal exhibits a pronouncedly higher non-sink dissolution plateau compared to the amorphous free from. This strikingly different behavior of both co-crystal forms for the compound under investigation, despite utilizing structurally very similar co-formers, may be explained by various contributions:

- As shown by Good et al. [40], there is a solubilizing effect of the co-former on the solubility of co-crystals, showing that co-crystal solubility is often proportional to the solubility of the respective co-former. Based on the higher solubility data of 2,4-DHBA versus 3,4-DHBA in organic solvents, as shown before, this may also facilitate higher supersaturation levels in aqueous media.
- As shown by Chen et al. [41], competitive processes between the attraction of taurocholate from the dissolution medium versus the attraction of water to crystal surfaces play a critical role in understanding the dissolution and supersaturation behavior in co-crystals. According to Chen et al., precipitation of the free form without the build-up of supersaturation levels may occur if a very fast selective dissolution of the co-former occurs from the co-crystal lattice, whereas a preferred coordination of taurocholate on the crystal faces can effectively slow down such a rapid dissolution of the co-former alone. Although no MD simulations are available for MSC178 solid-state forms and their interaction behavior with water and taurocholate, the pronouncedly stronger surface hydrophilicity in the 3,4-DHBA co-crystal compared to the 2,4-DHBA co-crystal (expressed in the higher total water-specific surface area equivalent as well as in the higher surface hydrophilic ratio), may explain the absence of any supersaturation effect in the non-sink dissolution compared to the 2,4-DHBA co-crystal. The 2,4-DHBA co-crystal is assumed to have a higher affinity for taurocholate molecule adsorption on the crystal faces upon the dissolution process, whereas for the 3,4-DHBA co-crystal, water adsorption is assumed to be more preferred, likely resulting in a fast release of the co-former and fast precipitation of the free form. The single-crystal structure of the 3,4-DHBA co-crystal indicates that the parallel chains formed by the API and the co-former, along with the hydrophilic channels running parallel to them, may facilitate the dissociation of the co-former pair more effectively than in the 2,4-DHBA co-crystal, where the DHBA homo-synthons are enclosed by API molecules. Over time, the API concentration is assumed to surpass its equilibrium solubility, prompting the transformation of the amorphous API into stable API crystals, in accordance with Ostwald’s law of stages. This process may lead to the precipitation of the crystalline API. Additionally, any excess 3,4-DHBA that exceeds its equilibrium solubility will also precipitate, which can cause the dissociation of the 3,4-DHBA co-crystal [42]. Our hypothesis is that in contrast to co-crystal form 34-1, a fast preferential dissolution of the co-former from the co-crystal lattice of form 24-1 and subsequent precipitation of the free form is suggested to be inhibited and slowed down due to the surface chemistry differences in co-crystal particles.
- The same hypothesis may explain the higher and more sustained supersaturation level in the 2,4-DHBA co-crystal compared to the 1,2-EDSA salt form, the latter also exhibiting a much higher surface hydrophilicity. In this case, however, other mechanisms, such as the protonation effect of the API in the dissolution layer, can additionally contribute to the supersaturation effect of the salt. The exceptional low surface hydrophilicity in the 2,4-DHBA co-crystal is also highlighted in comparison with the free base amorphous form, which shows a higher surface hydrophilicity despite the lack of any polar co-former molecules in the structure. This suggests that this aspect is an essential driver explaining the sustained supersaturation effect of the 2,4-DHBA co-crystal form for the specific solid-state system under investigation.
- The overall dense packing arrangement in the crystal structure of the 2,4-DHBA co-crystal, including multiple interactions involving the 2,4-DHBA co-former molecules in the structure, counteracts a fast and selective dissolution process of the acid, whereas in the 3,4-DHBA co-crystal, the water-accessible void structures and proximity of the 3,4-DHBA co-former to these voids via the para-hydroxyl group may ease such a dissolution process of co-former molecules alone.
- Another hypothesis may be that a stronger interaction between API molecules and the co-former molecules may also persist in the solution phase of the 2,4-DHBA co-crystal (compared to the solution phase of the 3,4-DHBA co-crystal), which may potentially inhibit a direct nucleation and precipitation of the free API from the supersaturated solution state. Such solution–state interactions were, in principle, shown by NMR data supersaturation mechanisms for API–polymer interactions [43]; however, in the case of MSC178 co-crystals, the first NMR experiments revealed concentrated solutions in aqueous media that were far too low to detect any API signals. The same holds true for Raman spectroscopic investigations by a Transmission Raman spectrometer on the FaSSIF solutions, which did not yield any API signals due to insufficient concentration levels of dissolved API. Attempts to derive the solution interaction strength of API to co-former molecules for the 2,4-DHBA and 3,4-DHBA co-crystals were made based on thermal analysis data after melting (representing a liquidus phase without any solvent); however, such data would be somewhat artificial, as no competitive effect of the aqueous milieu and the taurocholate environment is reflected. Moreover, even bearing this in mind, observed degradation onsets from TGA heating scans in the liquidus phase (as a measure of liquidus-phase interaction strength) do not support a stronger interaction between API and 2,4-DHBA compared to the interaction between API and 3,4-DHBA. Hence, there is no direct evidence of a potentially stronger API–co-former interaction in the solution phase of the 2,4-DHBA co-crystal. The observed heat capacity differences between the 2,4-DHBA co-crystal and the 3,4-DHBA co-crystal may indicate slightly lower molecular mobility in the solid state and a lower solid-state entropy for the 2,4-DHBA co-crystal; however, these are borderline insignificant with respect to the observed magnitude of differences. Nevertheless, the heat-capacity differences may be seen as supportive trends in combination with the higher melting point in the 2,4-DHBA co-crystal form 24-1, suggesting stronger lattice interactions between the API and co-former in this form. This may contribute to a stronger initial interaction in the diffusion layer, potentially also strengthened by the aromatic interactions between the API and 2,4-DHBA, as observed in the crystal structure. Overall, however, these findings regarding potential differences in interaction strength between co-former and MSC178 molecules are rather weak to substantially explain the observed dissolution plateau differences.
- The lower surface hydrophilicity in the 2,4-DHBA co-crystal form 24-1 compared to the co-crystal form 34-1 is also suggested by the crystal morphologies calculated by the BFDH method, showing that mainly hydrophobic functional groups are present in the dominant crystal faces of the 2,4-DHBA co-crystal form 24-1 (pyrazole and fluoropyridines), whereas polar hydroxyl groups are present in the main crystal faces of the 3,4-DHBA co-crystal form 34-1. It must be mentioned that the BFDH method predicts crystal morphology based solely on crystallographic parameters and does not account for factors such as surface chemistry, growth kinetics, solvent effects, or defects. Without experimental data like SEM or optical microscopy to validate these predictions, the BFDH results remain highly approximate and may not accurately reflect the real crystal habits observed. Therefore, conclusions drawn solely from BFDH predictions should be considered with caution.

The comprehensive characterization data of both identified co-crystals (2,4-DHBA co-crystal versus 3,4-DHBA co-crystal) show that to attain high-supersaturating co-crystals, it is not sufficient to focus solely on reducing the lattice energy by disrupting crystal lattice interactions (i.e., to overcome “brick-stone”-like properties), but adequate solubilizing behavior in the quaternary interaction API–co-former–bio-surfactant (taurocholate)–water must also be considered. Our hypothesis is that, to a certain extent, a stronger co-former coordination within the co-crystal lattice may even be beneficial to avoid too fast and selective co-former dissolution from the co-crystal matrix.

In contrast to the 2,4-DHBA co-crystal, the 1,2-EDSA salt undergoes a phase conversion in the suspension vehicle. Although the stronger *pH* shift impact upon dissolution in the diffusion layer of the 1,2-EDSA salt, triggering a higher supersaturation and subsequently precipitation of the free form, may strongly contribute to this conversion (as discussed in Section 2.3.2), additional contributions might be derived from differences in the surface chemistry of crystal faces in the 1,2-EDSA salt compared to the crystal faces in the 2,4-DHBA co-crystal. Sorption data confirm that the 2,4-DHBA co-crystal exhibits a pronouncedly lower water-specific surface area equivalent and surface hydrophilic ratio compared to the 1,2-EDSA salt. It is suggested that the competitive process on the particle surface between water and excipient molecules from the vehicle (methocel, tween) may also play a role in the stability behavior in the suspension vehicle. A stronger affinity of associated crystal faces in the 2,4-DHBA co-crystal to the less hydrophilic excipient molecules (relative to the adsorption affinity of water molecules) can be assumed, compared to the 1,2-EDSA salt, which bears more hydrophilic surface domains. The higher water affinity on crystal surfaces in the 1,2-EDSA salt may facilitate dissociation in the surface layers, which could act as seeds for adjacent layers in crystals. Interestingly, the high water affinity in the 1,2-EDSA salt is not an intrinsic feature of the presence of the protonated salt form, as the 3,4-DHBA co-crystal also exhibits a higher surface hydrophilic ratio than the 2,4-DHBA co-crystal. Although water activity and affinity to water vapor uptake on solids were demonstrated as being directly correlating factors with disproportionation effects in salt forms [44], the findings for the 3,4-DHBA co-crystal suggest that surface hydrophilicity is not per se driven by the presence or absence of ionic interactions in the crystal lattice but rather a consequence of the concrete surface chemistry in the most dominant crystal faces in each solid-state form.

The small g-scale upscale of the 2,4-DHBA co-crystal revealed a low extent of anisotropic growth in primary particles. This can be explained by the extended multi-dimensional hydrogen bonding network as well as the additional aromatic interactions present in the 2,4-DHBA co-crystal structure. At the molecular level, multiple options persist for dissolved molecules to attach to the surfaces of first nuclei and crystals. We believe that this may also explain the high tendency for the formation of intergrown dendrite-like secondary particles, as orthogonal growth directions can be realized when attaching via different molecular coordination motives. The obtained particle sizes from the combination of primary and secondary particle growth are in an ideal window to avoid sedimentation in the suspension vehicle (if too large particles) and to avoid agglomeration (if too small particles). Moreover, the fractal particle surface structure in the intergrown secondary particles may help avoid agglomeration (less particle-to-particle contact area) and is assumed to be beneficial in avoiding pronounced surface wettability to trigger dissociation (as also seen in low total water-specific surface area equivalent). It must be emphasized that these favorable particle properties are already achieved without any crystallization process optimization, and further options are possible to additionally gain control over the crystallization process with respect to primary particle size distribution and particle shape (e.g., by more elaborately defined seeding strategies). Even without further optimization, the favorable secondary particle properties are anticipated to result in good powder processing properties (such as good flowability, high bulk density), highlighting the potential of co-crystal forms also in the context of improving manufacturability aspects [45]. The g-scale manufactured 2,4-DHBA co-crystal and its inherent particle properties would allow usage in more sophisticated formulation vehicles, such as Powder-In-Capsules, for higher-species animal PK studies.

The in vivo PK data corroborate the physical chemistry profile of the different solid forms that were characterized and described in the previous sections of this paper, showing that the 2,4-DHBA co-crystal form 24-1 of MSC178 is stable in the oral methocel/tween vehicle used in the study. Although based on very limited datasets, there is an interesting (however, rather qualitative) match between the observed non-sink dissolution profile differences in the biorelevant intestinal medium (FaSSIF) of the 2,4-DHBA co-crystal form 24-1 versus the amorphous form of MSC178 and the observed PK exposure differences in both forms (2.4-fold in vivo exposure increase in the co-crystal form 24-1 versus the amorphous free form [based on *AUC*_0–24 h_ data in the PK study] in comparison with 2.3-fold in vitro supersaturation increase in the co-crystal form 24-1 versus the amorphous free form [based on *AUC*_0–120 min_ data in the non-sink dissolution profiles]).

The overall limited number of replicates in most characterization data (most often limited to *n* = 2, *n* = 3, or *n* = 4 replicates per solid-state form for quantitative methods due to limitations in available sample quantities) prevents a comprehensive statistical assessment of data and establishment of correlations. Nevertheless, the reported data and differences in characterization data between solid-state forms still suggest a meaningful differentiation of solid-state form characteristics, especially for surface characterization data, non-sink dissolution behavior, and in vivo PK exposure.

It is interesting to observe that the predicted molecular hetero-dimer excess enthalpies based on COSMOquick calculations for both co-crystal forms (2,4-DHBA co-crystal form 24-1, 3,4-DHBA co-crystal form 34-1) match in rank order with the experimentally observed dissolution/supersaturation effects (i.e., the more negative the calculated excess enthalpy, the higher the supersaturation behavior). Although this may, on first glimpse, be counterintuitive, this was also observed in the literature for COSMO-RS predictions (interaction strength API–co-former) in combination with MD calculations (mean-distance API–co-former in aqueous media), overall postulating that co-formers with high-excess energy in predicted co-crystal interactions can effectively compete with water for interactions with API and inhibit nucleation [46]. The data presented in the current study indicate that a far less complex approach utilizing the simplified excess enthalpy assessment based on the COSMOquick fragmentation approach could potentially also be useful in identifying co-formers to achieve supersaturation effects. However, more comprehensive and systematic work is needed to substantiate this hypothesis.

Moreover, internally developed algorithms for co-crystal formation likelihood were examined on the system. These algorithms combine computational chemistry to calculate interaction features, such as excess enthalpy, free energy of mixing, shape fitting parameters and solubility of the API in the co-former, with machine learning algorithms trained on a sufficient experimental database, yielding a score on the co-crystal formation likelihood ranging from 0 to 1 [47]. These calculations show an even more discriminating effect between the 2,4-DHBA and 3,4-DHBA co-crystals for MSC178 (the predicted co-crystal formation score is a factor of 2.5 higher for the 2,4-DHBA co-crystal than for the 3,4-DHBA co-crystal, compared to the factor 1.3 higher excess enthalpy for the 2,4-DHBA co-crystal versus the 3,4-DHBA co-crystal from COSMOQUICK). The higher score of 2,4-DHBA compared to 3,4-DHBA is related to the difference in excess enthalpy and differences in molecular descriptors, such as hydrogen bond acceptor strength, entropic contributions, chemical potential, and *σ*-profiles, in general. Although only observed for this specific pair of co-crystals of MSC178 so far, this indicates that the internally developed co-crystal prediction tool may also provide better discriminative power for co-crystal performance aspects, such as supersaturation.

The intrinsic design of the reported study resulted in data for a single research compound only, with a limited number of co-formers. While it is acknowledged that any kind of generalization or establishment of trends is not possible based on the reported data, this study does highlight some interesting key findings for the specific compound and respective solid-state forms under investigation, as outlined above. Nevertheless, more systematic work would be needed, and a study of corresponding data for other compounds and their respective solid-state forms is also encouraged to gain a more fundamental mechanistic understanding. Whereas the comparison of the co-crystal approach with alternative formulation strategies (such as amorphous solid dispersions, nanosuspensions, and co-amorphous systems) was not in scope for the current study due to initially discussed constraints, generally, a more systematic investigation on benchmarking co-crystal forms versus such alternative formulation approaches would also be desirable for a broader range of API structures as well (as partly shown for other formulation technologies in comparison with co-crystals by Li et al. [48] and Karagianni et al. [49]).

## 4. Materials and Methods

**Materials**

The API free base material for the crystal engineering study was obtained from Medicinal Chemistry laboratories, following synthetic routes, as described by Burgdorf et al. [7]. Different lab-scale batches from the same synthetic route were used for solubility and crystal engineering experiments, all with purity levels of at least 98% a/a (based on a generic HPLC method from synthesis labs).

High-purity p.A.-grade solvents were used for solubility and crystallization experiments (1,4-dioxane: Merck KGaA [Darmstadt, Germany] LiChrosolv, cat.-no. 1.03132; acetonitrile: Merck KGaA [Darmstadt, Germany] LiChrosolv cat.-no. 1.00030; ethyl acetate: Merck KGaA [Darmstadt, Germany] Emsure cat.-no. 1.09623; n-heptane: Merck KGaA [Darmstadt, Germany] LiChrosolv, cat.-no. 1.04390).

2,4-dihydroxybenzoic acid was obtained from SAFC (St. Louis, MO, USA; cat. no. 102698557, purity 97%). 3,4-dihydroxybenzoic acid was obtained from Thermo Scientific (Waltham, MA, USA; cat. no. B24016.14, purity 97%). 1,2-ethandisulfonic acid dihydrate was obtained from Thermo Scientific (Waltham, MA, USA; cat. no. 384800050, purity 98%). Hydrochloric acid was purchased from VWR (Darmstadt, Germany) as 32% aqueous solution (cat.-no. 20259.310) and diluted 1:10 in water prior to addition to crystallization trials.

Formic acid 98-100% was obtained from Merck KGaA (Darmstadt, Germany; Emprove® Essential Ph Eur cat.-no. 1002631000). Methocel ™ K4M was obtained from ChemPoint (Dublin, Ireland). Tween® 20, Molecular Biology grade, was purchased from Sigma-Aldrich (Sigma-Aldrich Chemie GmbH, Taufkirchen, Germany).

**In silico assessment of suitable co-formers for salts and co-crystals**

Excess enthalpy (*H_ex_*) calculations of molecular hetero-dimers, API:co-former, relative to molecular homo-dimers, API:API and co-former:co-former, were calculated in COSMOquick software (C. Loschen, A. Hellweg, A. Klamt, COSMOquick, version 1.3; COSMOlogic GmbH & Co. KG, Leverkusen, Germany, 2014). *H_ex_* levels were calculated at 298 K for a 1:1 stoichiometry.

**Solubility assessment of MSC178 and solid co-formers**

Solubility estimation of MSC178 and solid co-formers 1,2-EDSA, 2,4-DHBA, and 3,4-DHBA was performed at room temperature using the incremental solvent addition method. For this, defined quantities of respective solid material were accurately weighed into 1.5 mL glass vials with tight screw caps, and magnetic stirring bars were added. Vials were placed on a multi-position stirring plate at room temperature (20–25 °C) and agitated. Solvent was increment-wise added to the pre-weighted defined quantities of solid material in steps of 50 µL up to total volumes of 300 µL, and in steps of 100–250 µL until total max. volumes of 1.5 mL were reached. After each solvent addition step, the vials were tightly closed again, and equilibration of dispersions under stirring was allowed for at least 15 min. In the case that undissolved solid residues were visually identified after the solvent addition step, further solvent increments were added according to the above solvent addition process until a visually clear solution was obtained.

**mg-scale salt and co-crystal formation trials**

Approx. 15–20 mg of the starting material MSC178 (amorphous form) was dispersed into 1.5 mL ethyl acetate or 1.7 mL acetonitrile in 4 mL glass vials with tightly closed screw caps. Magnetic stirring bars were inserted into dispersions, and the vials were placed in a custom-made multi-position thermo-block, which was placed on a magnetic stirrer (IKA [Staufen, Germany] RCT basic). The thermo-block was attached to a programmable cryostat (Huber [Offenburg, Germany] petite fleur line). Dispersions were heated to 50 °C under gentle stirring to obtain solutions that were almost visually clear at 50 °C. Excess quantities of the respective solid co-former were used, reflecting the solubility ratio of MSC178 to co-former in the respective solvent. Respective calculated excess quantities of solid co-formers were weighed and added to the respective heated dispersions. In the case of HCl, approx. 1.1 eq. of stock solution made from a 1:10 dilution of 32% aqueous HCl solution was accurately pipetted to the warm solution of MSC178. Solutions or (in the case of spontaneous precipitation after addition of co-former) dispersions were cooled down from 50 °C to 4 °C in 10 h using a linear cooling ramp, followed by re-heating to 50 °C in 1 h. This cooling/heating cycle was repeated four times, followed by a final slurry agitation step at 4 °C for at least 2 h. Final suspension samples were solid/liquid-separated by centrifugation (Eppendorf [Leipzig, Germany] 5810R) at 12,000 rpm. Supernatant solutions were pipetted off and discarded. Wet solid residues were gently dried under nitrogen purge at room temperature for at least 1 h.

For miniaturized upscales, experiments were set up analogously using approx. 45–80 mg of the starting material MSC178 (amorphous form) and respective scaled larger quantities of solvent and co-formers. The 45 mg upscale trials were performed in 8 mL glass vials with tightly closed screw caps, using a second custom-made multi-position thermo-block with larger cavities to host the larger vials. For the edisylate salt and 3,4-DHBA co-crystal, slight variations in the stochiometric quantities used were adapted based on the results of co-former equivalents retrieved from solid samples of the initial 15 mg trials.

The experimental details of the utilized quantities in the mg-scale screening trials are summarized in Table 9.

**Process upscale of 2,4-DHBA co-crystal**

Upscaling of the 2,4-DHBA co-crystal was performed at the 1.7 g scale in an EasyMax crystallization platform in a 400 mL reactor and an overhead 3-bladed propeller stirrer with blades upwards (EasyMax 402, Mettler-Toledo GmbH, Giessen, Germany). A Mettler-Toledo (Giessen, Germany) ParticleTrack^TM^ (FBRM G400) probe and a Mettler-Toledo (Giessen, Germany) ParticleView^TM^ (PVM19) probe, as well as a Mettler-Toledo (Giessen, Germany) Pt100 temperature sensor, were immersed as PAT probes in the reactor through the PTFE reactor lid. First, 1.675 g of the MSC178 amorphous form was suspended in 185 mL of acetonitrile and agitated at 400 rpm. The suspension was heated in the EasyMax reactor to 50 °C and held for approx. 60 min, still yielding a slightly residual turbid suspension. Then, 4.178 g of 2,4-DHBA was added as a solid, and the suspension was heated to 60 °C at 10 K/min to obtain a clear solution, followed by a 30 min hold time at 60 °C. The solution was then cooled from 60 °C to 5 °C at 0.1 K/min. The final suspension obtained at 5 °C was agitated for a prolonged hold time for approx. 40 h to allow crystal growth. The suspension was filtered via a vacuum filtration unit with a cellulose paper filter. The wet filter cake was washed with approx. 10 mL n-heptane (p.A.-grade) and subsequently dried under nitrogen purge at room temperature.

Reaction control and data analysis of PAT trends were done in iControl software (ver. 6.0.53), interfaced with iC FBRM software (ver. 4.4.33) and iC PVM software (ver. 7.0.188), respectively (all from Mettler-Toledo GmbH, Giessen, Germany).

**Powder X-Ray Diffraction**

Samples (solids as well as suspensions from vehicle stability assessment) were prepared in a combinatorial 96-well plate (comprising an X-ray amorphous foil as bottom). Measurements were performed in transmission geometry with Cu-Kα1 radiation on a Stoe (Darmstadt, Germany) StadiP 611 diffractometer equipped with a Mythen detector. Scans were recorded from 0 to 36° 2θ simultaneously (step width of 0.03° 2θ; 30 s per step). Diffraction data were exported to Microsoft Excel to create diffraction pattern plots.

**Differential Scanning Calorimetry**

Samples were investigated on a Mettler-Toledo (Giessen, Germany) heat-flux Differential Scanning Calorimeter DSC1 with an autosampler, using a nitrogen inert gas atmosphere (50 mL/min). Overview scans were carried out in Al 40 µL pans with open lids from 25 to 250 °C at 5 °C/min (sample weights of 2–5 mg). Modulated DSC (mDSC) was performed in Al 40 µL pans (sample weights 3–8 mg) with centering pins and a laser-perforated 50 µm pinhole via Mettler-Toledo TOPEM^TM^ technology with stochastic temperature modulation, using a linear heating rate of 1 K/min from 25 to 75 °C, a temperature amplitude of 1 K, and a stochastic modulation phase between 15 and 45 s, respectively. Heat capacity profiles were calculated by reversing heat flow after Fourier Transformation in Mettler-Toledo (Giessen, Germany) STARe software ver. 16.0. The weights of the Al sample and reference pans were used as heat capacity references. Duplicate preparations were measured on two different samples of each solid-state form.

**Gravimetric Vapor Sorption (water vapor sorption)**

Samples were weighed into disposable Al pans and placed on the sample position of the GVS instrument with microbalance and incubator (DVS-Intrinsic, Surface Measurement Systems, SMS [Alperton, UK]). A nitrogen overall flow rate of 200 mL/min (combined dry and humid stream) was used for humidification. Milli-Q water from internal supply pipes was used in the water reservoir. Water vapor sorption isotherms were acquired at 25 °C, using an adsorption segment from 0% *rh* to 98% *rh* (with 10% *rh* steps and a final 8% *rh* step, respectively) and a final desorption segment from 98% *rh* to 0% *rh* (with an initial 8% *rh* step and 10% rh steps, respectively). The *rh* levels were set-up based on mass flow regulators. For all *rh* steps, an equilibrium condition of dm/dt ≤ 0.0005 wt%/min was used, with a minimum *rh* step time of 10 min and a maximum *rh* step time (timeout) of 360 min. Isotherms were calculated from the final weight readings at the end of each kinetic equilibration step and referenced to dry weight from the end of the 0% *rh* step. Sorption isotherms were calculated and plotted in Microsoft Excel using DVS Standard Analysis Suite (ver. 7.1.0.25) from SMS (Alperton, UK).

Additional water vapor sorption data were acquired with higher *rh* resolution in the lower partial pressure range, using an initial drying step at 0% *rh* for 240 min followed by adsorption segments 5–50% *rh* in steps of 5% *rh*. For all *rh* steps, an equilibrium condition of dm/dt ≤ 0.0005 wt%/min was used, with a minimum *rh* step time of 10 min and a maximum *rh* step time (timeout) of 360 min. Low partial-pressure isotherms were calculated acc. to the Excess Surface Work (ESW) sorption model, as described by Adolphs et al. [28], based on chemical potential changes Δ*µ* and the calculated Excess Surface Work (ESW) term φ upon the adsorption process:

(1) $$∆\mu =RT\cdot ln\frac{p}{p_{s}}$$

(2) $$\varphi ∶=n_{ads}\cdot ∆µ$$

ESW term φ was normalized twice by (a) dividing ESW by the molar quantity of sorbed water in the minimum of the ESW plot and (b) dividing normalized ESW by the product of universal gas constant R multiplied by temperature *T*:

(3) $$\varphi_{notm1}=\frac{\varphi }{n_{{ads}_{min}}}$$

(4) $$\varphi_{notm2}=\frac{\varphi_{norm1}}{RT}$$

These normalization procedures reduce the y-axis to a dimensionless scale that reveals the loss of degrees of freedom upon the sorption process.

For each normalized ESW plot, a minimum point is obtained. This minimum point can be interpreted as *rh*, where the strongest interaction of adsorbed water vapor with the powder surface is realized, hence resembling an equivalent of maximum primary surface coverage of water vapor (at a lower *rh* below the minimum point, full coverage of primary hydrophilic surface domains by water vapor is not yet realized; at a higher *rh* above the minimum point, increasing multilayer sorption decreases the overall sorption energy). Consequently, the adsorbed molar quantity in the minimum point of the normalized ESW function, $n_{{ads}_{min}}$, can be re-calculated as a water-specific surface area equivalent (to account for surface area domains that can realize maximum sorption energy upon adsorption of water vapor) using the Avogadro constant and the mean cross-sectional area of a single water molecule (10^−20^ m^2^/molecule). For each solid-state form under investigation, ESW sorption data were acquired from two measurements. The ESW sorption model was plotted and evaluated in Microsoft Excel based on exported GVS data from the measurement instrument.

**Organic Vapor Sorption (n-octane sorption)**

Samples were weighed into disposable Al pans and placed on the sample position of the OVS instrument with a microbalance and an incubator (DVS-Advantage, Surface Measurement Systems, SMS [Alperton, UK]). A nitrogen overall flow rate of 200 mL/min (combined dry and humid stream) was used for organic vapor generation. N-octane sorption isotherms were acquired at 25 °C in partial pressure (p/p_s_), range 0–50% with 10% p/p_s_ steps, using n-octane extra pure solvent (Acros Organics [Geel, Belgium], cat.-no. 129375000). Prior to the n-octane sorption steps, equilibration under dry nitrogen (0% *p/p_s_*) was performed for 240 min. N-octane levels were adjusted by feedback loop (closed-loop) from Dew Point Analyser (DPA) sensor readings. For all n-octane sorption steps, an equilibrium condition of dm/dt ≤ 0.0005 wt%/min was used, with a minimum step time of 10 min and a maximum step time (timeout) of 360 min. The resulting n-octane gravimetric sorption data were evaluated for n-octane-based specific surface area (*SSA*) acc. to the BET model in Microsoft Excel using DVS Advanced Analysis Suite (ver. 7.1.0.25) from SMS (Alperton, UK), in the partial pressure range 5–30% *p/p_s_* using a mean cross-sectional area of a single n-octane molecule of 63 Ă^2^/molecule [5, Williams].

**Single-Crystal X-Ray Diffraction**

Single crystals of the 2,4-DHBA co-crystal were selected and mounted on a XtaLAB Synergy R, HyPix-Arc 150 diffractometer (Rigaku, Oxford Diffraction, Tokyo, Japan). The crystal was kept at 99.9 (5) K during data collection. The instrument was operated, and the diffraction data were processed in the program CrysAlis^Pro^ [Rigaku Oxford Diffraction, CrysAlis^Pro^ software system, Rigaku Corporation, Wrocław, Poland, 2025 (version 1.171.44.117a)]. Using Olex2 [50], the structure was solved with the SHELXT [51] structure solution program using Intrinsic Phasing and refined with the SHELXL [51] refinement package using Least Squares minimization. All non-hydrogen atoms were refined through anisotropic displacement parameters, and all non-protic H-atoms were geometrically positioned and refined as riding. Protic H-atoms bound to heteroatoms were assigned by residual electron density. The CF_3_ group is disordered over two positions and was refined using a disorder model with respective occupancies of 0.57/0.43.

Crystal Data for the 2,4-DHBA co-crystal can be found in Table 10.

**Single-Crystal Structure Determination via 3D Electron Diffraction**

(General remarks) Electrons feature very strong interactions with the electrostatic potential of atoms. Subsequently, electron diffraction allows for performing experiments with crystallites in the nanometer range. However, it needs to be considered that the absorption of the samples is much stronger, and the data are affected by dynamical diffraction as well as ionic scattering factors compared to X-ray diffraction. This can lead to seemingly bad R-values for the refinement in the simplistic kinematic approximation.

Microcrystalline powder of the 3,4-DHBA co-crystal was spread on a lacey-carbon-supported copper TEM grid. Colorless crystallites with a few 100 nm thickness were selected for 3D-ED/microED measurements. Cryo-transfer, i.e., freezing samples prior to introduction to vacuum, at 100 K using a Gatan ELSA (Model 698) specimen holder was applied here.

Electron diffraction measurements were collected using an XtaLAB Synergy-ED (Rigaku corporation and JEOL Ltd., Tokyo, Japan), equipped with a Rigaku HyPix-ED detector optimized for operation in the continuous rotation 3D-ED experimental setup [52,53]. Data acquisition was performed at 100 K under high vacuum with an electron wavelength of 0.0251 Å (200 kV). The instrument was operated, and the diffraction data were processed in the program CrysAlis^Pro^ [Rigaku Oxford Diffraction, CrysAlis^Pro^ software system, Rigaku Corporation, Wrocław, Poland, 2025 (version 1.171.44.117a)]. A multi-scan absorption correction was performed using spherical harmonics implemented in the SCALE3 ABSPACK scaling algorithm in CrysAlis^Pro^. The structure was solved using ShelXT [49] and subsequently refined with kinematical approximation using ShelXL [49] in the crystallographic program suite Olex2 [Rigaku Oxford Diffraction, AutoChem 7 software system in conjunction with OLEX2, Rigaku Corporation, Wrocław, Poland, 2025 (version 1.5-ac7-018)]. By merging data of four individual grains/datasets, a completeness of 99.6% up to a resolution of 0.80 Å was achieved. All non-hydrogen atoms were refined through anisotropic displacement parameters, and all non-protic H-atoms were geometrically positioned and refined as riding. Protic H-atoms bound to heteroatoms were assigned by residual electron density. The CF_3_ group is disordered over two positions and was refined using a disorder model with respective occupancies of 0.57/0.43. The structure contains voids (8.1% of unit cell volume, 109.59 Å^3^ determined with 0.3 Å grid spacing and 1.2 Å probe size). Within these voids, some residual electron density was observed but could not be modeled. Due to the kinematical refinement, the electron number definition using solvent masking algorithms led to inaccurate results and was therefore avoided, leaving the residual electron density unassigned.

Crystal data for the 3,4-DHBA co-crystal (merged) can be found in Table 10.

**Non-sink miniaturized dissolution assessment**

Approx. 5 mg of a solid sample was weighed into glass vials. Then, 7 mL of FaSSIF (Biorelevant, London, UK) medium was added (after being pre-warmed to 37 °C), and the suspension was shaken at 450 rpm at 37 °C. After 5 min, 10 min, 15 min, 30 min, 60 min, and 120 min, 1 mL suspension was withdrawn and filtered through a 0.2 µm syringe filter (VWR, Darmstadt, Germany). The resulting clear filtrate was analyzed by UPLC (Waters GmbH, Eschborn, Germany) after suitable dilution to measure the amount of API dissolved, using the following UPLC method:

- Column: Acquity UPLC BEH C18 1.7 µm (2.1 × 50 mm);
- Solvent A: water/formic acid (999:1; *v*/*v*);
- Solvent B: acetonitrile/formic acid (999:1; *v*/*v*);
- Injection volume: 5 µL;
- Column temperature: 37 °CUPLC Gradient, as outlined in Table 11.

The calibration curve for determining the levels of dissolved API was established using a standard solution of free MSC178 with a known concentration. UV evaluation of all chromatograms was performed at 343 nm. All chromatographic data analysis was performed in Chromeleon software (ver. 7.2.10). Each form was analyzed in duplicate for every sampling timepoint.

**Vehicle stability assessment of lead forms**

Vehicle stability assessment was performed with the 2,4-DHBA co-crystal form 24-1 and the 1,2-EDSA salt form Ed-3 (samples from 50 mg upscale each). First, 2.5 mg of each form was weighed into a 1 mL glass vial. Then, 500 μL of vehicle (methocel 0.5% + Tween20 0.25% in water) was added, and the mixtures were treated with an Ultra Turrax device (IKA [Staufen, Germany]) at max. speed for 1 min. After preparation (t0) and after 24 h storage at room temperature, the suspensions were analyzed by Powder X-Ray Diffraction. The suspension after 24 h was also underwent visual examination and *pH* testing (*pH*-Meter 780, Metrohm GmbH, Filderstadt, Germany).

**In vivo mouse oral PK study of the 2,4-DHBA co-crystal and MSC178 free base**

Oral administration of the tested compounds (2,4-DHBA co-crystal form 24-1 [batch B-1] and amorphous MSC178 free base [batch A-2]) were performed at an equivalent dose of 50 mg/kg of the free base in methocel (0.5%)/tween 20 (0.25%) in water for injection by gavage at a dose volume of 10 mL/kg in female Crl:CD1(ICR) mice (Charles River Laboratories Models and Services, Sulzfeld, Germany) (*n* = 3 per group, housed together in cages with elevated grid). Blood samples (20 µL) were taken sublingually 0.25, 0.5, 1, 2, 4, 6, and 24 h after oral administration using K3-EDTA-coated capillaries (Microvette® 200, Sarstedt AG & Co. KG, Nümbrecht, Germany). Plasma was obtained by centrifugation (10,000 g; 4 °C; 5 min; Eppendorf [Leipzig, Germany] 5810R) and stored at −20 ± 5 °C until bioanalytical quantification by LC-MS/MS.

The plasma concentrations were quantified by UPLC with tandem mass spectrometric detection (UPLC-MS/MS) analysis, previously developed at Nuvisan GmbH. The UPLC-MS system consisted of a Waters Acquity UPLC (Waters GmbH, Eschborn, Germany) coupled to an AB Sciex mass spectrometer API 5500 Qtrap (AB Sciex Germany GmbH, Darmstadt, Germany). The UPLC methods used a reversed-phase column (Acquity UPLC HSS T3, 1.8 μM, 2.1 × 50 mm; Waters GmbH, Eschborn, Germany) and elution with a short gradient starting from 0.1% formic acid in water to 0.1% formic acid in acetonitrile (see the UPLC conditions, the gradient used, and the mass spectrometer conditions in the table below). Plasma samples (5 µL) were precipitated after the addition of 20 µL of internal standard solution (deuterated pruvanserine-d8) and 100 µL of acetonitrile. After centrifugation (10,000 g; 4 °C; 5 min; Eppendorf [Leipzig, Germany] 5810R), 50 µL of the supernatant was diluted with 100 µL water. An aliquot of 20 µL was further diluted with 180 µL of acetonitrile:water (1:9, *v*/*v*) prior to analysis.

A calibration curve was prepared for MSC178 with standard samples at different concentrations using blank rat plasma. The UPLC-MS/MS parameters are detailed in Table 12.

The maximum plasma concentration (*Cmax*) and time to reach the maximum plasma concentration (*tmax*) were obtained from the observed data. The custom-made software ‘DDSTOX’ developed at Nuvisan GmbH, which delivers comparable PK parameters to the validated software WinNonlin^®^ (Princeton, NJ, USA), was used to calculate the area under the plasma concentration–time curve (*AUC*). The *AUC* was obtained by non-compartmental analysis with the linear up/log down trapezoidal method.

## 5. Conclusions

Overall, the data in this study illustrate that a co-crystal strategy can be successfully developed in early pre-clinical industrial research with lean methodologies to optimize unfavorable phys.-chem. properties of a free base. The selected 2,4-DHBA co-crystal form had superior properties compared to an alternative co-crystal form with 3,4-DHBA as well as an edisylate salt form, which could be linked to the intrinsic features of the crystal structure and overall particle properties of the co-crystal phase. Excellent agreement between in vitro supersaturation behavior and in vivo exposure gain was demonstrated for the 2,4-DHBA co-crystal form, leading to better oral absorption in vivo and lower interindividual variability in exposure compared to the amorphous free base form.

## Figures and Tables

**Table 1:** Physicochemical properties of MSC178A (free form).

Parameter	Value
Molecular weight	431.35 g/mol
*HBD*/*HBA*	1/8
*pK_a_*	2.60 ^1^, 9.80 ^2^
log *P*	3.0 ^3^
log *D*	3.2 ^3^

^1^ Basic *pK_a_* (UV titration); ^2^ acidic *pK_a_* (UV titration); ^3^ measured (shake-flask method).

**Table 2:** Results of in silico prediction (COSMOquick) of toxicology-accepted co-formers.

Co-Former	Excess Enthalpy H_ex_	pK_a_ (ΔpKa versus MSC178) ^1^
Hydrochloric acid (HCl)	−3.3 kcal/mol	−7 (9.6) ^2^
3,4-Dihydroxybenzoic acid (3,4-DHBA)	−3.5 kcal/mol	4.16 (1.56) ^3^
2,4-Dihydroxybenzoic acid (2,4-DHBA)	−4.5 kcal/mol	3.10 (0.50) ^3^
1,2 Ethanedisulfonic acid (1,2-EDSA)	−5.5 kcal/mol	−1.45 (4.05) ^2^ −2.15 (4.75) ^2^

^1^ pKa values calculated with ChemAxon MarvinSketch Iodine.9 (version 21.15.9); ^2^ salt formation aimed for based on ΔpKa co-former versus MSC178; ^3^ co-crystal formation aimed for based on ΔpKa co-former versus MSC178.

**Table 3:** Solubility assessment (50 °C) in selected organic process solvents.

Solvent (Rel. Polarity ^1^)	Solubility MSC178A	Solubility 2,4-DHBA	Solubility 3,4-DHBA
acetonitrile (0.46)	~9 mg/mL	~29 mg/mL	~6 mg/mL
ethyl acetate (0.228)	~11 mg/mL	~55 mg/mL	>30 mg/mL
1,4-dioxane (0.164)	>46 mg/mL	>240 mg/mL	~85 mg/mL

^1^ Relative solvent polarities: hexane = 0.009; water = 1 [23].

**Table 4:** Summary of co-crystallizations (cooling crystallizations; 15 mg scale).

Co-Former	Solvent	Analytical Data Solid-State Residue		
		Crystallinity ^1^	Stoichiometry ^2^	Residual Solvents ^3^
HCl	acetonitrile	Crystalline, form Cl-1	0.9 eq. chloride	MeCN n.d.
	ethyl acetate	Crystalline, form Cl-2	0.9 eq. chloride	EtOAc n.d., acetic acid 1.0 eq.
1,2-EDSA	acetonitrile	Poorly crystalline, form Ed-1	0.7 eq. 1,2-EDSA	MeCN n.d.
	ethyl acetate	Crystalline, form Ed-2	0.7 eq. 1,2-EDSA	EtOAc 0.02 eq.
2,4-DHBA	acetonitrile	Crystalline, form 24-1	1.1 eq. 2,4-DHBA	MeCN n.d.
	ethyl acetate	Crystalline, form 24-2 ^4^	3.6 eq. 2,4-DHBA	EtOAc 0.4 eq.
3,4-DHBA	acetonitrile	Crystalline, form 34-1	1.3 eq. 3,4-DHBA	MeCN n.d.
	ethyl acetate	Crystalline, form 34-2 ^4^	2.1 eq. 3,4-DHBA	EtOAc 1.0 eq.

^1^ Powder X-Ray Diffraction; ^2 1^H-NMR or IC (chloride); ^3 1^H-NMR; ^4^ hints for signals of excess free co-former.

**Table 5:** Summary of optimized co-crystallizations (cooling crystallizations; 50 mg scale).

Co-Former	Solvent/ Target Stoichiometry	Analytical Data Solid-State Residue		
		Crystallinity ^1^	Stoichiometry ^2^	Residual Solvents ^3^
HCl	acetonitrile/1:1	Crystalline, form Cl-1	1.1 eq. chloride	MeCN 0.25 eq.
1,2-EDSA	ethyl acetate/1:1	Crystalline, form Ed-2	1.2 eq. 1,2-EDSA	EtOAc 0.007 eq.
1,2-EDSA	ethyl acetate/0.5:1	Crystalline, form Ed-3	0.6 eq. 1,2-EDSA	EtOAc 0.04 eq.
2,4-DHBA	acetonitrile/1:1	Crystalline, form 24-1	1.2 eq. 2,4-DHBA	MeCN n.d.
3,4-DHBA	acetonitrile/1:1	Crystalline, form 34-1	1.0 eq. 3,4-DHBA	MeCN n.d.

^1^ Powder X-Ray Diffraction; ^2 1^H-NMR or IC (chloride); ^3 1^H-NMR.

**Table 6:** Summary of surface area data derived for MSC178 1,2-EDSA salt, 2,4-DHBA co-crystal, and 3,4-DHBA salt versus amorphous free base (for comparison) (mean of *n* = 2 ± deviations single versus mean values from duplicate determination).

Solid-State Form	Water Vapor Sorption ESW Model	n-octane Sorption BET Model	Surface Hydrophilic Ratio ^1^
1,2-EDSA salt form Ed-3	water-specific surface area equivalent: 2.83 ± 0.21 m^2^/g	n-octane specific surface area: 8.38 ± 0.10 m^2^/g	0.34 ± 0.02
2,4-DHBA co-crystal form 24-1	water-specific surface area equivalent: 0.06 ± 0.00 m^2^/g	n-octane specific surface area: 0.52 ± 0.00 m^2^/g	0.11 ± 0.00
3,4-DHBA co-crystal form 34-1	water-specific surface area equivalent: 5.28 ± 0.02 m^2^/g	n-octane specific surface area: 24.38 ± 5.76 m^2^/g	0.23 ± 0.05
free base amorphous form	water-specific surface area equivalent: 4.51 ± 0.46 m^2^/g	n-octane specific surface area: 22.60 ± 5.30 m^2^/g	0.22 ± 0.07

^1^ water-specific surface area equivalent (ESW model)/n-octane surface area (BET model).

**Table 7:** Summary of upscale experiments of 2,4-DHBA co-crystal form 24-1 for PK studies.

Scale ^1^	Process Details	Analytical Data Solid-State Residue		
		Crystallinity ^2^	Stoichiometry ^3^	Residual Solvents ^4^
80 mg	▪ A total of ~83 mg API in 8 mL acetonitrile. ▪ Addition of ~220 mg 2,4-DHBA at 50 °C. ▪ Four temperature cycles of 50-5 °C at 0.075 °C/min. ▪ Filtration, drying at 50 °C (N2 purge).	Crystalline, form 24-1 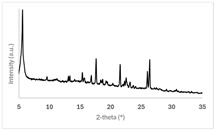	1.2 eq. 2,4-DHBA	MeCN 0.003 eq.
1.7 g	▪ A total of ~1.675 g API in 185 mL acetonitrile. ▪ Addition of ~4.178 mg 2,4-DHBA at 60 °C. ▪ Cooling ramp of 60–65 °C at 0.1 °C/min. ▪ Extended hold time at 5 °C for ~40 h ▪ Filtration, washing with n-heptane, drying at 50 °C (N2 purge).	Crystalline, form 24-1 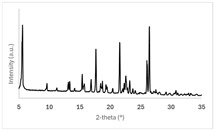	1.0 eq. 2,4-DHBA	MeCN 0.0007 eq./n-heptane ^5^ 0.0005 eq.

^1^ Free base quantity; ^2^ Powder X-Ray Diffraction; ^3 1^H-NMR or IC (chloride); ^4 1^H-NMR; ^5^ washing medium.

**Table 8:** Mouse oral high-dose PK data for the MSC178 amorphous free form versus the crystalline 2,4-DHBA co-crystal (suspension vehicle methocel/tween [0.5% methocel, 0.25% tween-20 in water]; 5 mg/mL ^1^; 50 mg/kg ^1^; mean data from *n* = 3 animals for each form).

Solid-State Form	tmax	Cmax	AUC_α_ ^2^
free base amorphous form	6 h	1750 ng/mL	32,800 h×ng/mL
2,4-DHBA co-crystal form 24-1	0.5 h	5290 ng/mL	77,500 h×ng/mL

^1^ Amounts refer to the free base entity in all cases; ^2^ 0–24 h, *AUC* to infinity is not reported because of large (>20% of measured *AUC*) extrapolated *AUC*.

**Table 9:** Experimental details of mg-scale co-crystallization experiments.

Co-Former	Solvent/ Target Stoichiometry	Quantities to Set-Up Experiments		
		MSC178	Solvent	Co-Former
HCl	ethyl acetate/1:1	15.8 mg	1.5 mL	40 µL 1:10 dilution
	acetonitrile/1:1	15.8 mg	1.7 mL	40 µL 1:10 dilution
	acetonitrile/1:1	46.9 mg	5.2 mL	131 µL 1:10 dilution
1,2-EDSA	ethyl acetate/1:1	16.4 mg	1.5 mL	9.2 mg
	acetonitrile/1:1	16.3 mg	1.7 mL	9.0 mg
	ethyl acetate/1:1	48.1 mg	4.5 mL	35.9 mg
	ethyl acetate/0.5:1	49.8 mg	4.5 mL	17.8 mg
2,4-DHBA	ethyl acetate/1:1	15.7 mg	1.5 mL	51.2 mg
	acetonitrile/1:1	16.2 mg	1.7 mL	42.0 mg
	acetonitrile/1:1	45.8 mg	5.0 mL	134.5 mg
	acetonitrile/1:1	83.0 mg	8.0 mL	220 mg
3,4-DHBA	ethyl acetate/1:1	17.3 mg	1.5 mL	62.3 mg
	acetonitrile/1:1	16.9 mg	1.7 mL	13.2 mg
	acetonitrile/1:1	48.4 mg	5.0 mL	23.5 mg

**Table 10:** Crystal data and structure refinement for the 2,4-DHBA and 3,4-DHBA co-crystals.

	2,4-DHBA Co-Crystal	3,4-DHBA Co-Crystal
Empirical formula	C_26_H_19_F_4_N_7_O_5_	C_26_H_19_F_4_N_7_O_5_
Formula weight	585.48	585.48
Temperature [K]	99.9 (5)	100 (5)
Crystal system	Triclinic	Triclinic
Space group	*P*-1	*P*-1
*a, b, c* [Å]	8.3301 (4), 10.0638 (4), 15.9928 (7)	4.6424 (4), 17.1020 (13), 17.2070 (8)
*α, β, γ* [°]	80.955 (4), 77.931 (4), 84.053 (4)	87.353 (5), 87.609 (6), 84.408 (7)
Volume [Å^3^]	1291.30 (10)	1357.23 (17)
*Z*	2	2
*D*_calc_ [g/cm^3^]	1.506	1.433
*µ* [mm^−1^]	1.095	0.000
F(000)	600	216
Crystal size [mm^3^]	0.15 × 0.03 × 0.02	nanocrystals
Radiation	Cu Kα (λ = 1.54184)	electron (λ = 0.0251)
*2Θ* range for data collection	5.706 to 149.866	0.084 to 1.798
Index ranges	−10 ≤ h ≤ 8, −11 ≤ k ≤ 12, −19 ≤ l ≤ 19	−5 ≤ h ≤ 5, −21 ≤ k ≤ 21, −21 ≤ l ≤ 21
Reflections collected	20,556	26,423
Independent reflections	5057 [R_int_ = 0.0352, R_sigma_ = 0.0407]	5535 [R_int_ = 0.1741, R_sigma_ = 0.1218]
Data/restraints/parameters	5057/4/424	5535/1274/421
Goodness-of-fit on F^2^	1.034	1.121
Final R indexes (I ≥ 2σ (I))	R_1_ = 0.0461, wR_2_ = 0.1109	R_1_ = 0.1912, wR_2_ = 0.4553
Final R indexes (all data)	R_1_ = 0.0662, wR_2_ = 0.1197	R_1_ = 0.2292, wR_2_ = 0.4789
Largest diff. peak/hole (eÅ^−3^)	0.19/−0.28	0.35/−0.43
CCDC	2540221	2540222

**Table 11:** Gradient used in the UPLC method for non-sink dissolution level quantification.

Time [min]	Eluent A [%]	Eluent B [%]	Flow Rate [mL/min]
0	90	10	0.83
0.83	10	90	0.83
1.19	10	90	0.83
1.2	90	10	0.83

**Table 12:** UPLC-MS/MS method used in oral PK bioanalysis: UPLC and mass spectrometer conditions and gradient used.

UPLC Conditions			
Column:		HSS T3, 1.8 μm, 2.1 × 50 mm	
Column Temperature:		60.0 °C	
Autosampler Temperature:		10.0 °C	
Injection Volume:		3–5 μL	
Mobile Phase A:		Acetonitrile	
Mobile Phase B:		0.1% Formic acid in water	
Gradient			
Time [min]	Eluent A [%]	Eluent B [%]	Flow rate [mL/min]
0	1	99	0.95
1.0	90	10	0.95
1.2	85	15	0.95
1.3	1	99	0.95
Mass Spectrometer Conditions			
Scan Type:		MRM	
Polarity:		Positive	
Resolution *Q1*:		Unit	
Resolution *Q3*:		Unit	
Curtain Gas (CUR):		22.0 psi	
Spray Voltage:		4000 V	
Source Temperature:		650 °C	
Ion Source Gas 1(nebulizer gas):		65.0 psi	
Ion Source Gas 2 (heater gas):		65.0 psi	
Collision Gas (CAD):		Medium	
Entrance Potential (EP):		10.0 V	

## Data Availability

Supporting data on the reported results may be provided upon request from the authors. Cambridge Crystallographic Data Centre (CCDC) contains the supplementary crystallographic data for this publication, as submitted under CCDC codes 2540221 and 2540222. This data can be obtained free of charge via www.ccdc.cam.ac.uk/data_request/cif, by emailing data_request@ccdc.cam.ac.uk, or by contacting The Cambridge Crystallographic Data Centre, 12 Union Road, Cambridge CB2 1EZ, UK; fax: +44-1223-336033.

## Supplementary Materials

The following supporting information can be downloaded at https://www.mdpi.com/article/10.3390/ph19050803/s1, Figure S1: Water vapor data from MSC178 1,2-EDSA salt, 2,4-DHBA co-crystal, and 3,4-DHBA salt for assessment of surface area domains; Figure S2: n-octane vapor data of MSC178 1,2-EDSA salt, 2,4-DHBA co-crystal, and 3,4-DHBA salt for assessment of surface area domains; Figure S3: PAT data of g-scale upscale experiment of 2,4-DHBA co-crystal form 24-1; Figure S4: Chord length distribution (CLD) at end of 5 °C holding time of g-scale upscale experiment of 2,4-DHBA co-crystal form 24-1.

## Abbreviations

The following abbreviations are used in this manuscript:

PK	pharmacokinetic
MSC	Merck internal code for small-molecule compounds
POLθ	polymerase theta enzyme
COSMO/COSMO-RS	conductor-like screening model/ conductor-like screening model under real solvation
2,4-DHBA	2,4-dihydroxybenzoic acid
3,4-DHBA	3,4-dihydroxybenzoic acid
1,2-EDSA	1,2-ethanedisulfonic acid
mg	milligram
FDA	Food and Drug Administration
NCE	new chemical entity
PROTAC	proteolysis targeting chimera
i.v.	intravenous
SEDDS	self-emulsifying drug delivery system
ASD	amorphous solid dispersion
SDD	spray-dried dispersion
HME	hot-melt extrusion
MMEJ	microhomology-mediated end-joining
DDR	DNA damage and repair
DNA	deoxyribonucleic acid
NHEJ	non-homologous end-joining
HR	homologous recombination
FA	Fanconi anemia
*pK_a_*	ionization constant
log *P*	partition coefficient octanol/water or buffer (fully un-ionized species)
log *D*	distribution coefficient octanol/phosphate buffer pH 7.4
HBD	hydrogen bond donor
HBA	hydrogen bond acceptor
p.o.	per os
PEG	polyethylene glycole
HPB	hydroxypropylbetadex
mL	milliliter
DCS	development classification system
PAT	process analytical technology
g	gram
*H_ex_*	excess enthalpy
eq.	equivalents
HCl	hydrochloric acid
n.d.	not determined
IC	ion chromatography
^1^H-NMR	proton nuclear magnetic resonance spectroscopy
°C	degree Celsius
PXRD	powder X-ray diffraction
DSC/mDSC	differential scanning calorimetry/ temperature-modulated differential scanning calorimetry
*C_p_*	heat capacity (at constant pressure)
J	Joule
K	Kelvin
n	number of replicates in case of multiple determinations
*rh*	relative humidity
BET	Brunauer–Emmett–Teller (adsorption model)
ESW	Excess Surface Work (adsorption model)
BFDH	Bravais–Friedel–Donnay–Harker (crystal morphology model)
FaSSIF	fasted-state simulated intestinal fluid
min	minutes
*pH*	negative decadic logarithm of molar proton concentration
ICH	International Committee for Harmonization
CLD	chord length distribution
N2	nitrogen gas
*Cmax*	maximum observed concentration
*t-max*	timepoint at maximum observed concentration
*AUC*	area under the curve
MD	molecular dynamics
cat.-no.	catalogue number
FBRM	focused beam reflectance measurement (PAT technology)
PVM	particle view microscopy (PAT technology)
p.A.	pro analysi (analytical grade)
DPA	dew point analyser
GVS	gravimetric vapor sorption
DVS	dynamic vapor sorption
OVS	organic vapor sorption
UPLC	ultrahigh performance liquid chromatography
UV	ultraviolet
