# Supplementary material for: Crystal Engineering as an Efficient Medicinal Chemistry Tool for Animal PK Bioavailability Enhancement in Early Pre-Clinical Research
Source: Pharmaceuticals (Basel). 2026 May 21;19(5):803. doi: 10.3390/ph19050803 (PMC13210109; doi:10.3390/ph19050803)
Supplement: Supplementary file 1 [file pharmaceuticals-19-00803-s001.zip › pharmaceuticals-4251677-supplementary.pdf]

Supplementary Materials

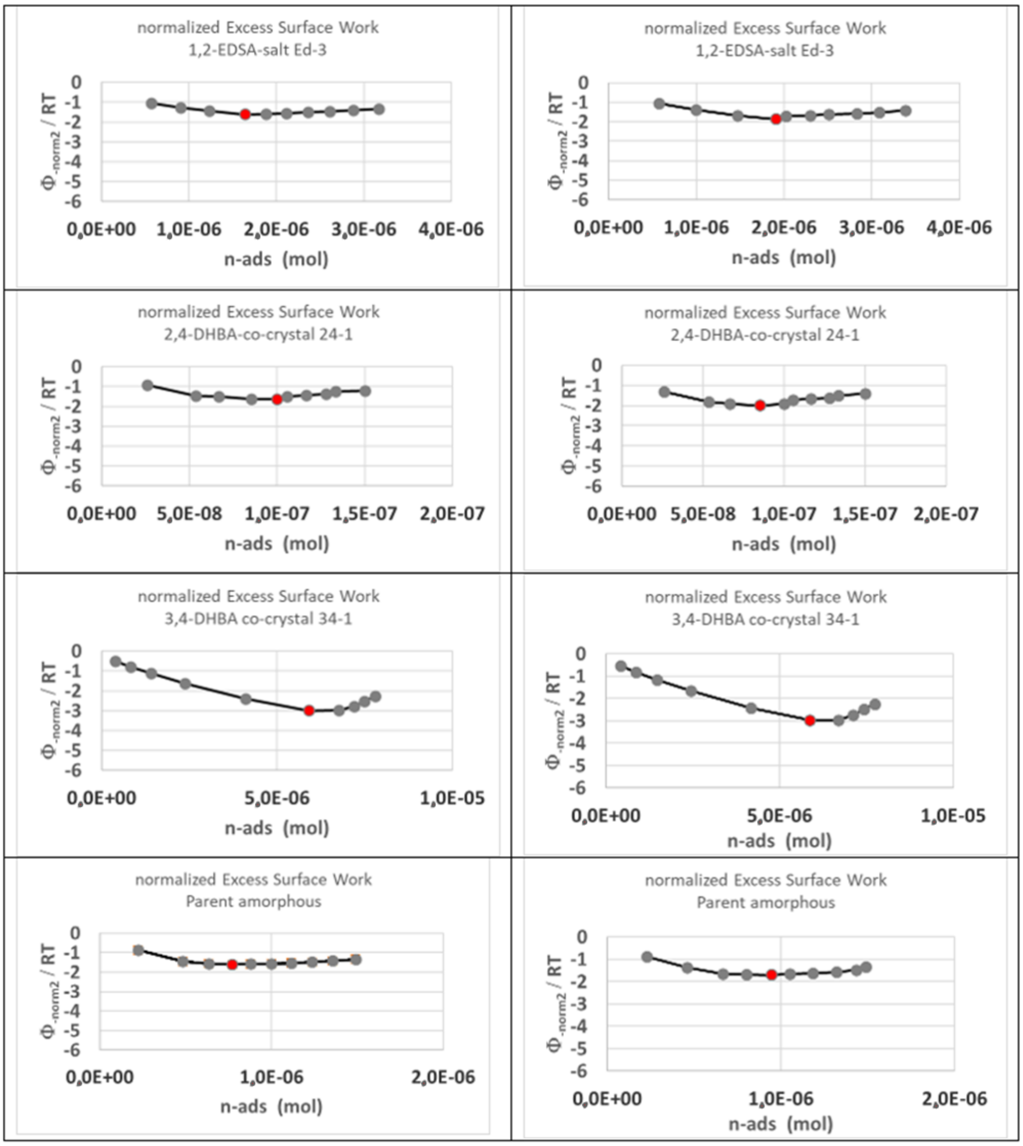

Figure S1. Water vapor data of MSC178 1,2-EDSA salt, 2,4-DHBA co-crystal, and 3,4-DHBA salt for assessment of surface area domains (partial pressure steps of 5% from 0-50% partial pressure). ESW model, 2 runs (ESW minimum point shown in red). (**top**): 1,2-EDSA salt form Ed-3; (**2nd from top**): 2,4-DHBA co-crystal form 24-1: (**2nd from bottom**): 3,4-DHBA co-crystal form 34-1; (**bottom**): amorphous free base

n-octane BET plot, 1,2-EDSA salt form Ed-3:

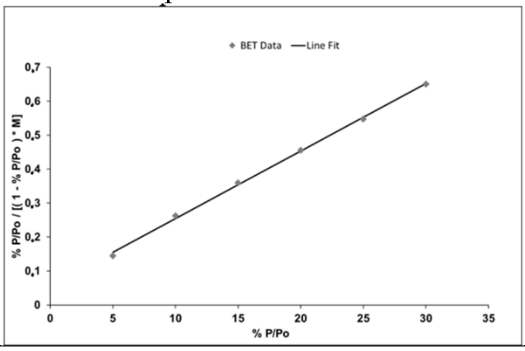

n-octane BET plot, 1,2-EDSA salt form Ed-3:

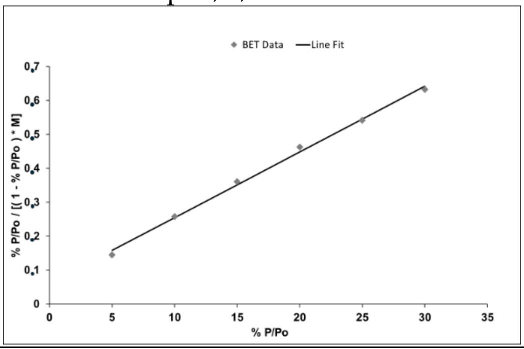

n-octane BET plot, 2,4-DHBA co-crystal form 24-1:

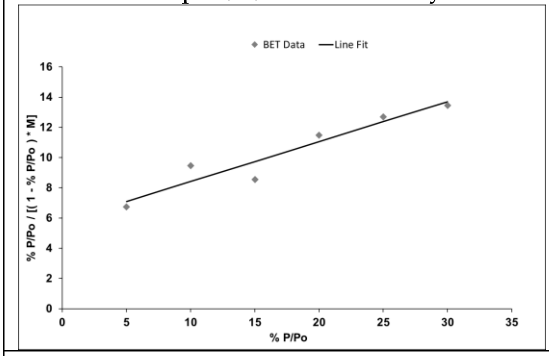

n-octane BET plot, 2,4-DHBA co-crystal form 24-1:

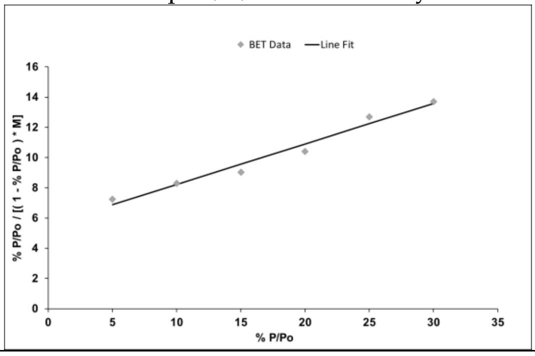

n-octane BET plot, 3,4-DHBA co-crystal form 34-1:

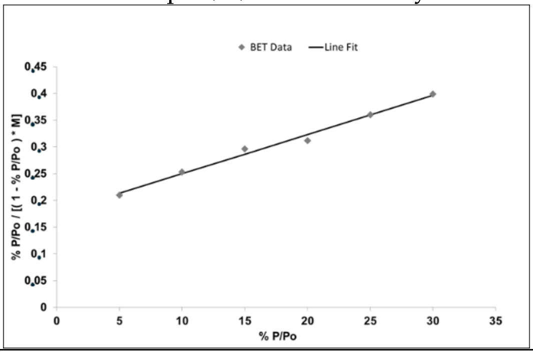

n-octane BET plot, 3,4-DHBA co-crystal form 34-1:

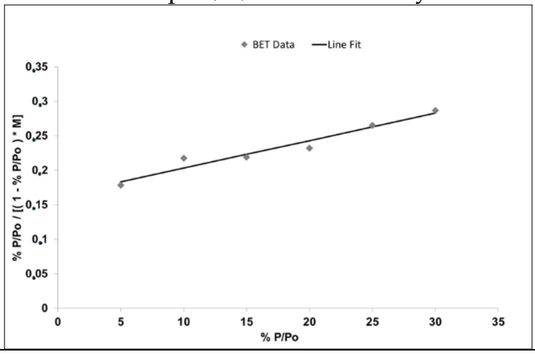

n-octane BET plot, amorphous free base:

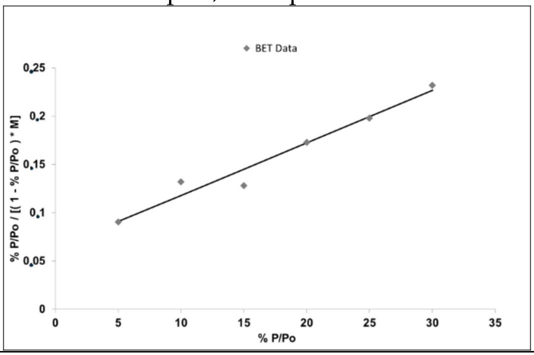

n-octane BET plot, amorphous free base:

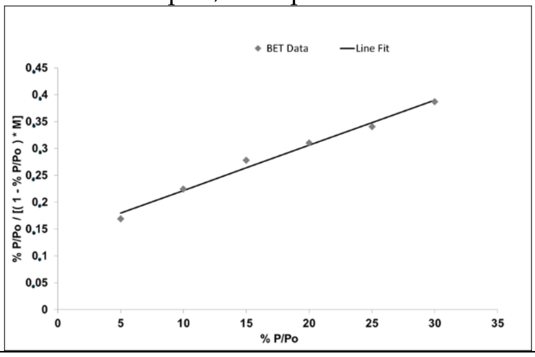

Figure S2. n-octane vapor data of MSC178 1,2-EDSA salt, 2,4-DHBA co-crystal, and 3,4-DHBA salt for assessment of surface area domains (partial pressure steps of 5% from 0-50% partial pressure). BET model, 2 runs. (**top**): 1,2-EDSA salt form Ed-3; (**2nd from top**): 2,4-DHBA co-crystal form 24-1: (**2nd from bottom**): 3,4-DHBA co-crystal form 34-1; (**bottom**): amorphous free base

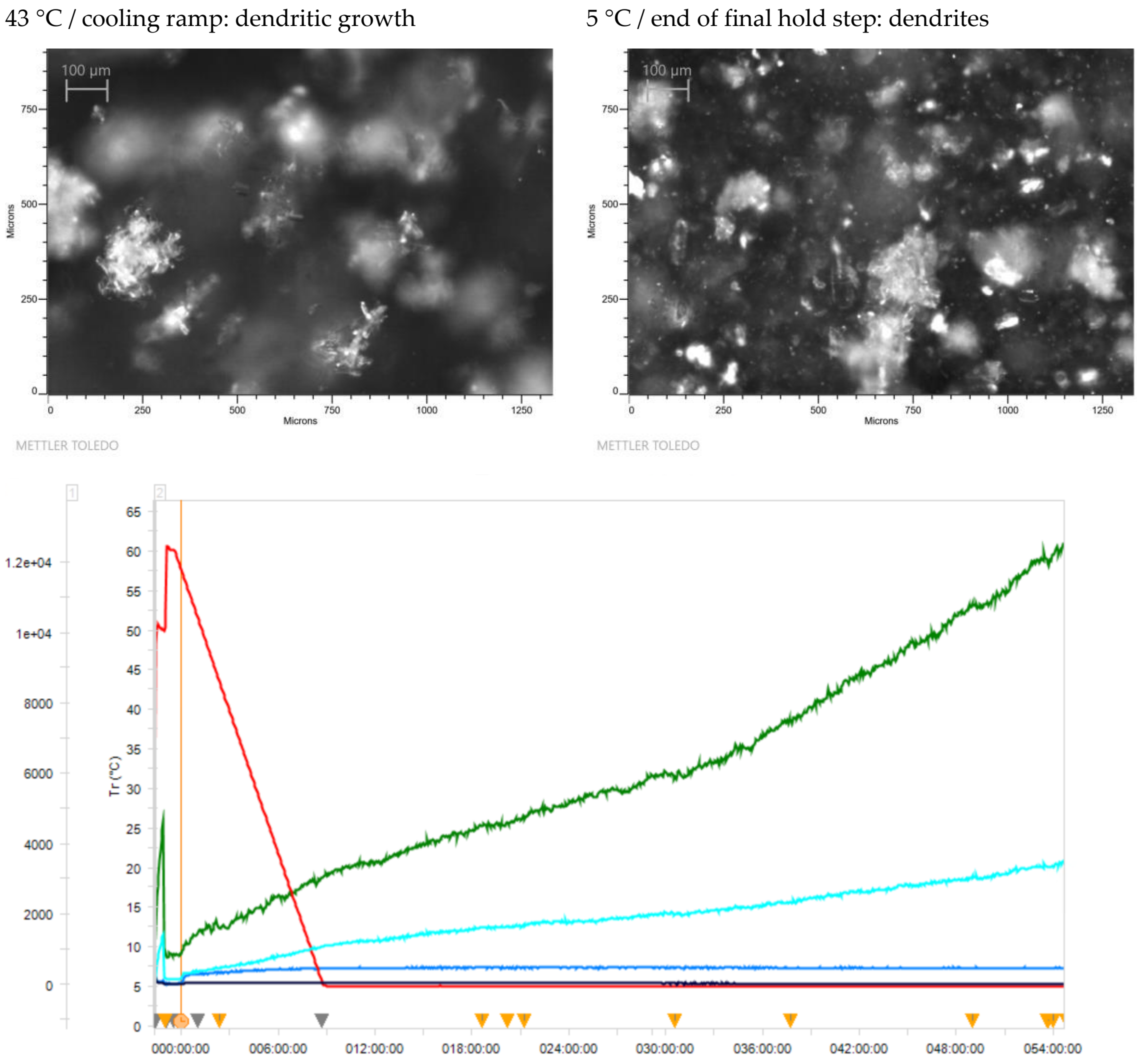

Figure S3. PAT data of g-scale upscale experiment of 2,4-DHBA co-crystal form 24-1; (**top**): PARTICLEVIEW™ images at various stages of crystallization process; (**bottom**): reaction temperature (red) vs particle counts from PARTICLETRACK™ (green: total counts, light blue: counts 10-50 µm, blue: counts 50-150 µm, dark blue: counts 150-300 µm)

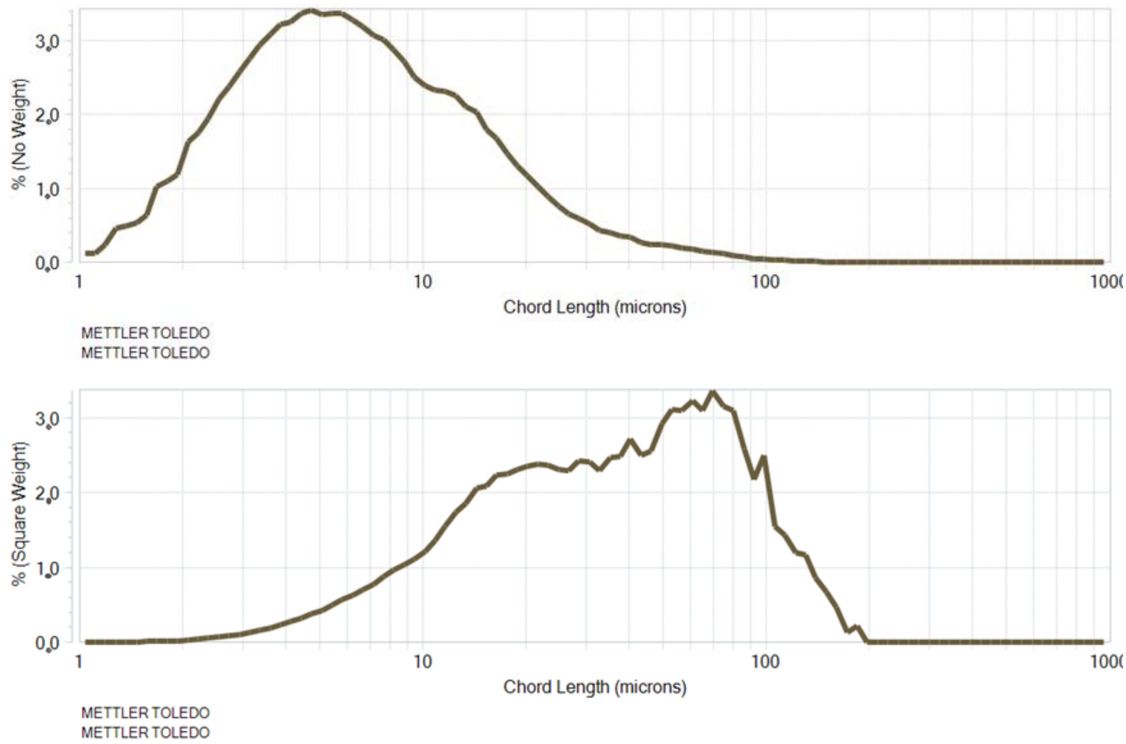

Figure S4. Chord Length Distribution (CLD) at end of 5 °C holding time of g-scale upscale experiment of 2,4-DHBA co-crystal form 24-1; (**top**): non-weighted; (**bottom**): square-weighted
